# Off-Label Use of Bumetanide for Brain Disorders: An Overview

**DOI:** 10.3389/fnins.2019.00310

**Published:** 2019-04-24

**Authors:** Shivani C. Kharod, Seok Kyu Kang, Shilpa D. Kadam

**Affiliations:** ^1^Neuroscience Laboratory, Hugo W. Moser Research Institute at Kennedy Krieger, Baltimore, MD, United States; ^2^Department of Neurology and Neurosurgery, Johns Hopkins University School of Medicine, Baltimore, MD, United States

**Keywords:** bumetanide (BTN), Na-K-Cl cotransporter 1 (NKCC1), neonatal seizures, autism, schizophrenia, temporal lobe epilepsy (TLE)

## Abstract

Bumetanide (BTN or BUM) is a FDA-approved potent loop diuretic (LD) that acts by antagonizing sodium-potassium-chloride (Na-K-Cl) cotransporters, NKCC1 (SLc12a2) and NKCC2. While NKCC1 is expressed both in the CNS and in systemic organs, NKCC2 is kidney-specific. The off-label use of BTN to modulate neuronal transmembrane Cl^−^ gradients by blocking NKCC1 in the CNS has now been tested as an anti-seizure agent and as an intervention for neurological disorders in pre-clinical studies with varying results. BTN safety and efficacy for its off-label use has also been tested in several clinical trials for neonates, children, adolescents, and adults. It failed to meet efficacy criteria for hypoxic-ischemic encephalopathy (HIE) neonatal seizures. In contrast, positive outcomes in temporal lobe epilepsy (TLE), autism, and schizophrenia trials have been attributed to BTN in studies evaluating its off-label use. NKCC1 is an electroneutral neuronal Cl^−^ importer and the dominance of NKCC1 function has been proposed as the common pathology for HIE seizures, TLE, autism, and schizophrenia. Therefore, the use of BTN to antagonize neuronal NKCC1 with the goal to lower internal Cl^−^ levels and promote GABAergic mediated hyperpolarization has been proposed. In this review, we summarize the data and results for pre-clinical and clinical studies that have tested off-label BTN interventions and report variable outcomes. We also compare the data underlying the developmental expression profile of NKCC1 and KCC2, highlight the limitations of BTN’s brain-availability and consider its actions on non-neuronal cells.

## Introduction

Bumetanide is a fast-acting LD, acting on the widely distributed NKCC1 (Slc12a2), and renal-specific NKCC2. LDs act on the loop of Henle and are often used clinically for palliative treatment of renal insufficiency, heart failure, nephrotic syndrome, and hypertension (Wittner et al., 1991; Brater, 2000). Patients that were prone to seizures but administered LDs to induce diuresis for these previously mentioned indications, reported notable anti-seizure effects (Hesdorffer et al., 1996; Hesdorffer et al., 2001; Kanner, 2002; Maa et al., 2011). While various mechanisms for the seizure alleviation were proposed, the use of loop-diuretics as anti-seizure drugs remains under investigation.

In the brain, the Cl^−^ importer NKCC1 is balanced by the function of Cl^−^ extruder, potassium-chloride-cotransporter 2 (KCC2). Increased expression of NKCC1, not balanced with the efflux action of KCC2, has been the rationale behind administration of BTN as an antiseizure agent. BTN has been administered for HIE neonatal seizures (NCT01434225, 2015; NCT00830531, 2017), but was reported inefficacious (Pressler et al., 2015). BTN administered to patients with autism (Lemonnier and Ben-Ari, 2010; Lemonnier et al., 2012, 2017), schizophrenia (Rahmanzadeh et al., 2017) and TLE (Eftekhari et al., 2013), however, reported beneficial effects.

The developmental expression profile of BTN’s primary target, NKCC1 (Morita et al., 2014; Sedmak et al., 2016), has recently been elucidated. Studies conducted to analyze BTN’s BBB penetration (Puskarjov et al., 2014), interaction with efflux transporters (Donovan et al., 2014, 2016; Römermann et al., 2017), blood plasma-binding properties (Donovan et al., 2016), diuresis (Asbury et al., 1972; Maa et al., 2011) and pharmacokinetic (PK) properties (Puskarjov et al., 2014) all have addressed questions about BTN’s brain availability. Of interest are BTN’s possible interactions with NKCC1 in non-neuronal cells (Zhang et al., 2014).

## Maintaining the Transmembrane Cl^−^ Gradient

**FIGURE 1:**
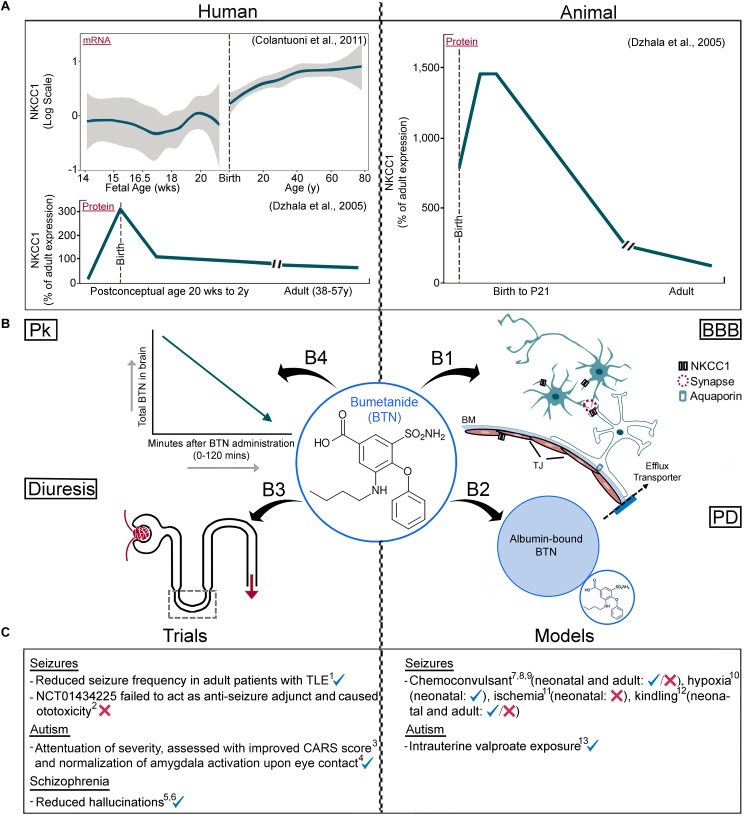
Parsing information about NKCC1 isoforms and BTN **(A)** Human vs. rodent NKCC1 expression profiles. mRNA data from microarray probe (hH034657, Illumina Technologies - probe chosen specifically because it spans most of NKCC1) shows developmental upregulation, followed by NKCC1a protein data using western blotting techniques showing a developmental downregulation. Rodent data of NKCC1a protein shows a developmental downregulation, quantitated with western blotting techniques and consistent with human data analyzed with western blotting using the same probes. **(B)** Pharmacological attributes of BTN affecting its direct neuronal modulation; **(B1)** BBB, neuronal and non-neuronal NKCC1, **(B2)** Albumin’s high affinity to BTN, **(B3)** BTN’s diuretic effects, **(B4)** BTN’s short half-life in serum and brain. **(C)** Clinical trials and pre-clinical models with varying results of BTN efficacy, see Table 1 for further details. Pk, pharmacokinetics; BBB, Blood–Brain Barrier; PD, pharmacodynamics; BM, basement membrane; TJ, tight junction. Panel A data adapted from Dzhala et al., 2005 with permission and Colantuoni et al., 2011 (graphed brain cloud data replotted with R statistical software).

Cl^−^ cotransporters, NKCC1 and KCC2, are the primary mediators that maintain neuronal transmembrane Cl^−^ gradient (Rivera et al., 1999; Ben-Ari et al., 2012; Côme et al., 2019). NKCC1 is expressed in multiple cell types in the CNS, including neurons, contributing to Cl^−^ intracellular accumulation (Dzhala et al., 2005; Nicholls et al., 2012). KCC2 expression, while thought to be neuronal specific (Song et al., 2002; Zhang et al., 2014), has been found outside the CNS as well (Antrobus et al., 2012), and extrudes Cl^−^ to maintain lower [Cl^−^]_i_. These two co-transporters mediate the GABA “switch,” and their functions contribute to inhibitory actions of GABA_A_ receptor (GABA_A_R) agonists (Owens and Kriegstein, 2002; Lee et al., 2005). Excitation/inhibition imbalance has been attributed to the developmental profiles of NKCC1 and KCC2 protein expression (Ben-Ari et al., 2012). In the immature brain and in certain pathological states, activation of GABA channels leads to the efflux of Cl^−^ ions due to high [Cl^−^]_i_, resulting in membrane depolarization. Achieving a balance between NKCC1 (Cl^−^ influx) and KCC2 (Cl^−^ efflux), by curbing excessive NKCC1 function has been the reasoning behind the off-label use of BTN, both in pre-clinical models and clinical studies (see Table 1 for details). Despite being a potent NKCC1 antagonist, BTN can also antagonize KCC2 at higher concentrations (Delpire et al., 2009). The developmental upregulation of KCC2 has been elucidated and confirmed with a wide span of experimental techniques (Uvarov et al., 2013; Sedmak et al., 2016). The expression profile of KCC2 correlates with maturation of different brain regions (Watanabe and Fukuda, 2015; Côme et al., 2019). The KCC2b isoform is developmentally upregulated, but KCC2a expression remains steady over brain maturation (Uvarov et al., 2007, 2009; Côme et al., 2019). Until recently, however, the developmental profile of NKCC1 isoforms has remained uncertain, mainly due to experimental limitations (Morita et al., 2014; Figure 1).

**Table 1:** BTN off-label studies.

	Study	Model	Strain	Age	Sex as a biological variable	BTN dose	Number of BTN doses	Dose delivery	Experimental paradigm	Reported effect
**Pre-clinical rodent studies (*in vivo*)**										
Neonatal seizures	Dzhala et al., 2005	KA	Long–Evans rats, Wistar rats and C57 mice	(P9–12), (P5–23), and (P7–9), respectively	EEG: M (Wistar), sex not specified for Long-Evans or C57	0.1–0.2 mg/kg (*in vivo*); 10 μM *in vitro*	1 *in vivo* and bath-applied *in vitro*	IP, *in vivo* and bath-applied *in vitro*	Bath applied post-elevated K+ *(in vitro)* and Injected 15 min post-KA *(in vivo)*	Epileptiform activity in hippocampal slices *in vitro*↓; KA-induced seizure’s *in vivo* ↓ EEG power
Neonatal seizures	Mares, 2009	PTZ	Wistar rats	P7, P12, P18	M	0.2, 0.5, 1, and 2.5 mg/kg	1	*In vivo*, IP	Pretreatment 20 min before PTZ *(in vivo)*	Dose-dependent effect in P12 (anticonvulsant at 1 mg/kg, and proconvulsant at 2.5 mg/kg); No effect in P7/P18.
Neonatal seizures	Mazarati et al., 2009	Rapid kindling	Wistar rats	P11, P14, P21	M	0.2, 0.5, or 2.5 mg/kg	1.5	*In vivo*, IP	Once upon detection of ADT ( + 1/2 dose during kindling procedure	Anticonvulsant at P11, no effect at P14/P21
Neonatal Sseizures	Liu et al., 2012	Right carotid ligation	Sprague–Dawley rats	P7	M/F	2.5 and 10 mg/kg	1	*In vivo*, IP	10 min after PB injection, which was administered 15 min post-hypoxia	Anticonvulsant effect together with PB (BTN: 10 mg/kg), no effect with 2.5 mg/kg BTN
Febrile seizures	Koyama et al., 2012	Hyperthermia	Sprague–Dawley rats	P11	M	0.1 mg/kg *in vivo*	6	IP, *in vivo* and bath-applied *in vitro*	Once daily from P11–P17 *in vivo* post-hyperthermia on P11	Rescue of granule cell ectopia, limbic seizure susceptibility and development of epilepsy
Neonatal seizures	Cleary et al., 2013	Hypoxia	Long–Evans rats	P10	M	0.15 or 0.3 mg/kg	1	*In vivo*, IP	15 min prior to seizure induction by hypoxia	Reversal of seizure-induced changes in E_GABA_ when compared to PB and/or BTN applied alone
Neonatal seizures	Kang et al., 2015	Right carotid ligation	CD-1 mice	P7, P10	M/F	0.1–0.2 mg/kg	1	*In vivo*, IP	1 h post-PB, 2 h post- unilateral carotid ligation	No effect/seizure aggravation at P10
Neonatal seizures	Wang et al., 2015	Hypoxia	Wistar rats	P10	Not indicated	0.5 mg/kg/day	21	*In vivo*, IP	Daily for 3 weeks post-hypoxia	Alteration of newborn DG cell structure and ↓ spontaneous EEG seizure’s after HI
Neonatal seizures	Holmes et al., 2015	Flurothyl	Sprague–Dawley rats	Induced seizure’s P5–14, tested for developmental alterations from P18–25	M	0.5 mg/kg	10	*In vivo*, IP	Twice daily, once before first flurothyl-induced seizure and again after the last seizure each day	Normalization of voltage correlation, sociability and seizure threshold
Neonatal seizures	Hu et al., 2017	PTZ after HI (Rice-Vanucci method)	Sprague–Dawley rats	P7	Unsexed	0.5 mg/kg	6	*In vivo*, IP	Twice daily for 3 days after surgery	PTZ-induced seizure susceptibility ↓, restoration of hippocampal neurogenesis, improved cognitive function
Neonatal seizures	Kharod et al., 2018	PTZ	CD-1 mice	P7	M/F	0.1–0.2 mg/kg	1	*In vivo*, IP	1 h post-PB, 2 h post-PTZ	No effect/seizure aggravation post-PB suppression in P7 females
TLE	Brandt et al., 2010	Pilocarpine	Sprague–Dawley rats	Adult	F	Three dosing protocols: (1) 0.2 mg/kg, (2) 10 mg/kg, (3) 0.8 mg/kg/h	(1) Multiple doses first 24 h, then 14, (2) multiple doses first 24 h, then 14, (3) continuous	(1) *In vivo*, IP (2) *in vivo*, IP (3) *in vivo*, IV	(1) First 24 h all 3–7 h, then twice daily for 2 weeks, (2) first 24 h all 3–7 h, then twice daily for 2 weeks, (3) continuous infusion after bolus of 2 mg/kg/ for 5 days	Combined PB/BTN treatment altered behavior consequences of epileptic rats
TLE	Sivakumaran and Maguire, 2016	KA	C57BL/6 mice	Adult	M	0.2 mg/kg or 2.0 mg/kg, i.p. *(in vivo)*, and 54.8 μM *(in vitro)*	1 *in vivo* and bath applied *in vitro*	IP, *in vivo* and intrahippocampal administration *in vitro*	BTN 30, 60, 90, and 120 min prior KA administration (*in vivo*) and direct hippocampal injection of BTN 30 min prior to KA injection (*in vitro*)	↓ KA-induced ictal activity *in vivo* and SLEs *in vitro*, restoration of diazepam efficacy *in vitro* and *in vivo*
TLE	Kourdougli et al., 2017	Pilocarpine	Wistar rats	Adult	M	86 ng/day	Continuous	*In vivo*, Osmotic minipumps	Continuous infusion for 3 days	Restored post-SE NKCC1/KCC2, normalized Cl^−^ homeostasis, ↓ of glutamatergic recurrent mf sprouting in DG
Autism	Tyzio et al., 2014	Rats exposed *in utero* to valproate (VPA rats) and mice carrying the Fragile X mutation (FRX mice)	Wistar rats, mice strain not specified	E18, P0, P2, P4, P7, P8, P15 and P30 (mice); E20, P0, P2, P4, P7, P15, and P30 (rats)	M/F	2–2.5 mg/kg *(in vivo)*, 10 μM *(in vitro)*	1	In drinking water (*in vivo*) + *in vitro*	BTN pretreatment – given to dams in drinking water (*in vivo*) and bath-applied *in vitro*	Maternal pretreatment restored electrophysiological and behavioral phenotypes in pups
Stroke	Xu et al., 2017	Endothelin stroke model	Wistar rats	Adult	M	0.2 mg/kg/day	Continuous	*In vivo*, IV; mini-osmotic pumps	21 days - continuous infusion	Enhancement of neurogenesis and behavioral recovery, no effects on inflammation
Periventricular leukomalacia	Jantzie et al., 2015	Unilateral carotid artery ligation followed by hypoxia	Long–Evans rats, protein-enhanced green fluorescent protein transgenic mouse pups (B6/CBA background)	P6	M	0.3 mg/kg	6	*In vivo*, IP	Every 12 h for 60 h post-HI	Attenuation of myelin base protein loss and neuronal degeneration 7 days post-HI
TBI	Lu et al., 2006	Weight drop device	Wistar rats	Adult	M	15 mg/kg	1	*In vivo*, IV	20 min before TBI	↓ Brain contusion volume
TBI	Lu et al., 2007	Weight drop device	Wistar rats	Adult	M	15 mg/kg	1	*In vivo*, IV	20 min before TBI	Attenuation of inflammatory response and neuronal loss
Neuropathic pain	Mòdol et al., 2014	Sciatic nerve injury	Sprague–Dawley rats	Adult	F	30 mg/kg	16	*In vivo*, IP	Days 1–16 - post injury	Prevented spinothalamic tract projecting area changes and hyperalgesia
Intracerebral hemorrhage	Wilkinson et al., 2019	Collagenase	Sprague–Dawley rats	Adult	M	10 and 40 mg/kg	Multiple doses and treatment groups	*In vivo*, oral and IP	2 h or 7 days post-ICH, either 6 or 12 h interval orally or IP for 3 days	Minor ↓ in edema after early dosing, no effect on behavior or injury volume, no normalization of ion concentration after late dosing
**Pre-clinical rodent studies (*in vitro* only)**										
Neonatal seizures	Dzhala et al., 2008	Low Mg^2+^	Sprague–Dawley rats	P4–P7	M	10 μM	Bath-applied	*In vitro*	Bath-applied after 5–8 recurrent ictal-like episodes	Efficacious adjunct to PB, ↓ recurrent tonic-clonic epileptiform activity
Febrile seizures	Reid et al., 2013	Lipopolysaccharide/KA + behavioral febrile seizure	Long–Evans rats	P14	M	10 μM	Bath-applied	*In vitro*	Bath-applied 30 min after application of 4-AP	↓*In vitro* 4-AP-induced inter-ictal activity in the inflammation and inflammation + FS groups
TLE	Bragin et al., 2009	Pilocarpine	Wistar rats	Adult	M	10 μM	Bath-applied	*In vitro*	Bath-applied; 20 min superfusion 3 weeks post-SE	Restoration of IPSP reversal potential and ↓ polysynaptic burst discharge
Schizophrenia and autism	Amin et al., 2017	22q11.2 DS hippocampal neurons	C57BL/6 J mice	Neurons from E18	Not indicated	10 (uM	Applied to cell culture media	In vitro	Applied to cell culture media at 16 DIV, then after 16 DIVs ( + baseline spiking activity	( hyperexcitable action of GABA_A_ receptor signaling, restored network homeostatic plasticity in *Lgdel*^+/-^ networks
**Off-label clinical studies**										
Neonatal seizures	Kahle et al., 2009	Human (case report)	n/a	6 weeks	F	0.1 mg/kg	1	IV	Single dose, post-PB and fosphenytoin	↓ Mean seizure duration and frequency
Neonatal seizures	NCT01434225, 2015	Human	n/a	Gestational age of 37–43 weeks and postnatal age <48 h	M/F	0.05, 0.1, 0.2, or 0.3 mg/kg	4	IV	Up to four times, 12 h intervals	No anticonvulsant effect, ototoxicity
Neonatal seizures	NCT00830531, 2017	Human	n/a	Post-conceptual age of 33–44 weeks	M/F	0.1, 0.2, or 0.3 mg/kg	1	IV	One dose together with PB after establishing PB-resistance with a first-line PB only dose	Results and summary statement on clinical trials.gov awaited
TLE	Eftekhari et al., 2013	Human	n/a	Adult – 31, 32, and 37 years	M	2 mg/day	Long-term administration	Oral	∼3/4 months + pre-existing anti-epileptic drugs	Seizure frequency ↓, epileptiform discharges ↓ on pre-vs. post EEG in 2 out of 3 patients
Autism	Lemonnier and Ben-Ari, 2010	Human	n/a	Age span from 3 years and 8 months to 11 years and 5 months	M/F	1 mg/day	Long-term administration	Oral	0.5 mg twice a day for 3 months	Improvement in IAS with no side effects
Autism	Lemonnier et al., 2012	Human	n/a	6.8 years ± 13.2 months	M/F	1 mg/day	Long-term administration	Oral	0.5 mg twice a day for 3 months, followed by 1 month washout	Improved CARS, CGI and Autism Diagnostic Observation Schedule values
Autism	Du et al., 2015	Human	n/a	2.5–6.5 years	M/F	1 mg/day	Long-term administration	Oral	0.5 mg twice a day for 3 months	ABC and CGI scores improved when ABA training combined with BTN treatment, compared to ABA training alone
Autism	Lemonnier et al., 2017	Human	n/a	2–18 years	M/F	1.0, 2.0, and 4.0 mg/day	Long-term administration	Oral	0.5, 1.0, and 2.0 mg twice daily for 3 months	Improved CARS, SRS and CGI scores
Autism	Hadjikhani et al., 2018	Human	n/a	14.8–28.5 years	M/F	1 mg/day	Long-term administration	Oral	Once daily for 10 months	More eye contact, less amygdala activation
Schizophrenia	Lemonnier et al., 2016	Human (case report)	n/a	14 years	M	2 mg/day	Long-term administration	Oral	Once daily for 11 months	↓ Hallucinations
Schizophrenia	Rahmanzadeh et al., 2016	Human	n/a	55.9 ± 13.9 years	M/F	1 mg	Long-term administration	Oral	Twice daily for 2 months	No effect on PANSS scores/subscores or BPRS score
Schizophrenia	Rahmanzadeh et al., 2017	Human	n/a	38–67 years	M/F	1 mg	Long-term administration	Oral	Twice daily for 2 months	↓ Hallucinations
Parkinson’s disease	Damier et al., 2016	Human	n/a	( >50 years (n ( =4)	M/F	5 mg	Long-term administration	Oral	Once daily for 2 months	Improvement of PD motor symptoms in all four patients, improvement of gait and freezing in 2 of these patients
**Human *in vitro* studies**										
Neonatal seizures (tuberous sclerosis complex and focal cortical dysplasia)	Talos et al., 2012	Human, TSC cortical slices	n/a	Infancy through adulthood (1.4–57 years)	M/F	10 μM	Bath-applied	*In vitro*	Bath-applied with NBQX and DL-AP5	Suppression of PSC amplitude and frequency
TLE	Palma et al., 2006	Human, surgical resection from hippocampus and temporal neocortex injected into oocytes	n/a	Adult (27, 29, 41, and 43 years)	M/F	12 μM	Bath-applied	*In vitro*	Oocytes treated with BTN (3 h)	Shifted the E_GABA_ to more negative in oocytes injected with membranes from TLE hippocampal subiculum
Brain tumor related epileptogenesis	Conti et al., 2011	Human, membranes from peritumoral cortical tissues of epileptic patients injected into oocytes	n/a	Adult (21–67 years)	M/F	12 μM	Bath-applied	*In vitro*	Oocytes pretreated with BTN (2 h)	Abolished difference of depolarized E_GABA_ in oocytes injected with epileptic peritumoral cerebral cortex
Sturge–Weber Syndrome	Tyzio et al., 2009	Human, neurons from human pediatric SWS cortex *in vitro*	n/a	Infancy (6, 9, 13, and 14 months)	M/F	10 μM	Bath-applied	*In vitro*	Bath-applied	No prominent effects on epileptiform activity
Focal cortical dysplasia	Blauwblomme et al., 2018	Human, slices from resected tissue from patients with FCD	n/a	2.8–16.9 years; BTN tested in 12 slices from 7 patients	M/F	8 μM	Bath-applied	*In vitro*	Bath-applied	Suppressed IIDs in 9 of 12 slices, IIDs reappeared after washout. No effect in 1 case, and reduced frequency and amplitude in 2 cases of FCD Type 1c

*NBQX, 2,3-dihydroxy-6-nitro-7-sulfamoyl-benzo[f]quinoxaline-2,3-dione; ADT, after discharge threshold; ABA, applied behavior analysis; ABC, autism behavior checklist; BPRS, Brief Psychiatric Rating Scale; BTN, bumetanide; CARS, Childhood Autism Rating Scale; CGI, clinical global impressions; DIV, days in vitro; DG, dentate gyrus; DL-AP5, DL-2-Amino-5-phosphonopentanoic acid; EEG, electroencephalogram; F, female; FCD, focal cortical dysplasia; HI, hypoxia-ischemia; IAS, infantile autistic syndromes; ICH, intracerebral hemorrhage; IIDs, interictal discharges; M, male; mf, mossy fiber; IPSP, inhibitory post-synaptic potential; KA, kainic acid; PD, Parkinson’s disease; PTZ, pentylenetetrazole; PB, phenobarbital; PANSS, Positive and Negative Syndrome Scale; PSC, post-synaptic current; sz, seizure; SRS, Social Responsive Scale; SE, status epilepticus; SWS, Sturge–Weber Syndrome; TLE, temporal lobe epilepsy; TBI, traumatic brain injury; TSC, tuberous sclerosis complex; yrs, years.*

## Developmental Profile of Nkcc1 Isoform Expression in the Brain

The developmental expression profile of NKCC1 mRNA has been examined in postmortem human brains with RT-PCR and was found to be stable postnatally (Morita et al., 2014). This contrasts with the age-dependent reduction of NKCC1 protein expression levels reported with rodent and human western blotting data (Dzhala et al., 2005; Kang et al., 2015) (Figure 1A). NKCC1 mRNA has been shown to have predominant expression in ventricular and periventricular cell populations at embryonic and early neonatal ages in the brain (Li et al., 2002). Toward P0, there was no more significant increase in expression of mRNA but distribution within cortical layers changed from ventricular zones to cortical layers (Li et al., 2002). After mRNA splicing, NKCC1 results in two main isoforms, NKCC1a and NKCC1b, that span across many tissue types in humans, including the brain (Vibat et al., 2001). Commercially available, NKCC1 western blotting antibodies, used in many of the earlier pre-clinical and human studies, failed to detect NKCC1b, one of the two main isoforms of NKCC1 in the brain (Dzhala et al., 2005; Morita et al., 2014; Kang et al., 2015). The western blotting probes and antibodies targeted exon 21, found in NKCC1a, but not NKCC1b (Clayton et al., 1998; Vibat et al., 2001; Kaila et al., 2014; Morita et al., 2014; Puskarjov et al., 2014; Kang et al., 2015). Since NKCC1b is the predominant isoform expressed in the brain, (Vibat et al., 2001), the apparent downregulation of NKCC1 total protein in maturing brains could be attributed to this experimental artifact. Additionally, (Morita et al., 2014) identified multiple isoforms with different developmental profiles in the human brain. Along with NKCC1a and b, the mRNA expression of recently discovered NKCC1 transcripts, 1–27 (21a), 1–4a and 1–2a were identified and verified in the DLPFC (Morita et al., 2014). The mRNA expressions of NKCC1a, NKCC1b, and 1–4a were low during fetal development, increased after birth through adolescence and reached stable levels in adulthood. This is further supported in a study that evaluated NKCC1 mRNA in P5–P90 rats. While high at P5, there was a significant drop at P10 that then remained stable with advancing age. Since P7 in rodents is close to term birth in humans, this would indicate stable NKCC1 mRNA expression in neurons after birth (Lee et al., 2010). Transcript 1–2a decreased after birth but was stable throughout postnatal life (Morita et al., 2014). Additionally, qRT-PCR was utilized in another study to determine developmental expression of NKCC1 (Hyde et al., 2011). An upregulation was seen after birth before leveling off at 20 and 23 years of age in the DLPFC and hippocampus, respectfully. Isoform specific quantification was not conducted in this study, however, and probes utilized spanned exons 4–5.

Brain Cloud^TM^ is an open-access online tool^1^, containing genetic and epigenetic data from human prefrontal cortex postmortem brains (Colantuoni et al., 2011). Microarray conducted on human postmortem brain tissue showed NKCC1 mRNA expression profiles from post-conceptual ages through adulthood. These data has been quantitated with two Illumina microarray probes (hHA034657 and hHC001510, Brain Cloud Expression data) spanning the length of all introns and exons to quantitate both isoform transcripts. Both microarray probes show an increase in expression of NKCC1 mRNA as the brain matures (UCSC Genome Browser, Brain Cloud Expression Data). The developmental upregulation of NKCC1 mRNA via microarray mRNA expression data has been reported in many brain regions (Sedmak et al., 2016). In contrast, however, NKCC1 mRNA has been shown to be downregulated in rat neocortical neurons with advancing age (P1–21) when assessed with RT-PCR (Yamada et al., 2004). Other studies utilized *in situ* hybridization to demonstrate downregulation in visual cortex from P0 to P28 (Ikeda et al., 2003), cerebral cortex and hippocampus [Plotkin et al., 1997b (P0-adult); Shimizu-Okabe et al., 2002 (P1–P28)]. Different parts of the brain express different levels of NKCC1 at different developmental timepoints. With advancing age, the expressions of certain transcripts are different than of others. With the availability of probes that target different parts of NKCC1 mRNA sequence, it is confounding on how to properly assess isoform-specific developmental NKCC1 profile. To validate their NKCC1 knockout mouse model, one study utilized multiple probes like mouse, rabbit and goat SLC12a2 antibodies against total protein, C-terminus and N-terminus (Antoine et al., 2013). Revalidation of western blot data with NKCC1-isoform-specific antibodies that can help quantify both NKCC1 isoforms accurately in humans and rodents is needed. Monoclonal antibodies targeting both NKCC1 and NKCC2 are currently available (Developmental Studies Hybridoma Bank at the University of Iowa). While these antibodies cannot help clarify the developmental expression profile of multiple NKCC1 isoform proteins in the brain (Morita et al., 2014), their specificity has only been validated using NKCC1-knockout mouse brains (Deidda et al., 2015). Some studies have tried to tackle this issue by reporting NKCC1 mRNA and comparing it to KCC2 total protein to help evaluate simultaneous expression (Reid et al., 2013). No western blotting probe currently allows us to identify and quantitate each isoform of NKCC1 independently. Western blotting samples from different brain regions also contain empty blood vessels lined with ependymal tissue and glial cells, both of which express NKCC1, representing contamination to assertions about neuronal NKCC1 expression profiles. This would be especially true both in embryonic and neonatal developmental brain studies.

## Action in Non-Neuronal Cells

NKCC1 has a widespread distribution throughout the body (Vibat et al., 2001) and maintains cellular ionic homeostasis through electroneutral movement of ions across the membrane (Geck et al., 1980; Markadieu and Delpire, 2014). In the CNS, NKCC1 is also expressed in ependymal and glial cells (Plotkin et al., 1997a; Wu et al., 1998; Hubner et al., 2001; Kanaka et al., 2001; Yan et al., 2001a,b; Mikawa et al., 2002; Su et al., 2002; Wang et al., 2003; Sun, 2010). NKCC1, assessed with RNA-seq, shows higher concentration of transcripts in mature astrocytes (human ages 8–63) than fetal astrocytes (18 gestational weeks) (Zhang et al., 2014). BTN improved ischemic cerebral edema in the post-ischemic brain (Yan et al., 2001b; O’Donnell et al., 2004). This effect is perhaps through BTN’s actions on ependymal NKCC1 (Patyal and Alvarez-Leefmans, 2016).

Na-K-Cl co-transport is responsible for regulating K^+^ concentration gradient in astrocytes (Hertz, 1965; Walz, 1987). This function is especially crucial in attempts to avoid excessive K^+^ accumulation that occurs after astrocyte swelling in pathological conditions, such as ischemia and traumatic brain injury (TBI) (Kimelberg, 1992; Rutledge and Kimelberg, 1996; Walz, 2000). Additionally, NKCC1 is involved in control of extracellular Ca^2+^ ions (Lenart et al., 2004; Annunziato et al., 2013) and astrocytes regulate neuronal Ca^2+^ levels through Ca^2+^-dependent glutamate release (Parpura et al., 1994). When NKCC1 activity was ablated or pharmaceutically inhibited in astrocytes, filling of Ca^2+^ endoplasmic-reticulum Ca^2+^ stores in astrocytes was absent following oxygen/glucose deprivation and reoxygenation (Lenart et al., 2004). Astrocytes are active modulators of neuronal activity. The vital relationship between neurons and astrocytes that allows for proper brain homeostasis could be indicative of further neuronal regulation via astrocytes (Figure 1B1). This concept could underlie one of the multifactorial mechanisms of BTN responsiveness in the CNS due to the role of astrocyte pathologies in the different neurological diseases.

The relationship between aquaporin 4 (AQP4) and NKCC1 has been investigated in the CNS; AQP4 effluxes water in response to NKCC1 transporting water (Østby et al., 2009; Zeuthen, 2010; Nagelhus and Ottersen, 2013), indicating other possible sites for BTN mediated modulation. NKCC1 expressed in the mouse choroid plexus is the main contributor to cerebrospinal fluid production, through its water-translocating properties (Steffensen et al., 2018). While once thought to be a passive process, recent studies show NKCC1 plays an active role in producing nearly half of the brains daily quota of CSF through the choroid plexus (Steffensen et al., 2018). NKCC1 is also robustly expressed in oligodendrocytes and has a pivotal role in GABAergic functions (Plotkin et al., 1997b; Wang et al., 2003; Annunziato et al., 2013). Muscimol-induced activation of GABA_A_R’s resulted in reduced [Cl^−^]_I_, cell shrinkage and NKCC1 activity (Wang et al., 2003). In fact, NKCC1 is most robustly expressed in newly formed oligodendrocytes when compared to all other neural cell types including astrocytes, neurons, oligodendrocyte progenitor cells, myelinating oligodendrocytes, microglia and epithelia (Zhang et al., 2014). BTN increased neurogenesis and alleviated stroke-induced behavioral impairments in adult rats (Xu et al., 2017). For the following studies, (O’Donnell et al., 2005, 2006), authors utilized T4 hybridoma pan NKCC1 and NKCC2 antibody. Since endothelial cells in the brain solely express NKCC1 and not NKCC2, one can infer that the quantifications reported were of NKCC1 only. Authors, however, reported it as NKCC protein since probe was not NKCC1 specific. NKCC1 function in endothelial cells, lining CNS microvasculature are significantly controlled by endogenous factors like arginine vasopressin and estradiol, (O’Donnell et al., 2005, 2006). It was previously shown that antagonizing NKCC with BTN reduced edema formation in a rat stroke model (O’Donnell et al., 2004). Arginine vasopressin stimulates NKCC activity during ischemia and promotes edema formation (O’Donnell et al., 2005). Estradiol was shown to reduce activity of NKCC and decreased edema formation (O’Donnell et al., 2006). Therefore, NKCC1 antagonist, BTN, could play a significant role over brain activity even before it crosses the BBB.

Outside the CNS, NKCC1 is expressed in the epithelial cells of the inner ear (Delpire et al., 1999). Maintenance of homeostasis of inner ear fluids is crucial for proper functioning of the auditory organs, and the inner ear is known to be sensitive to systemic offsets (Juhn et al., 1991). NKCC1 is expressed in the inner and middle ear (Crouch et al., 1997; Kim et al., 2007; Abbas and Whitfield, 2009). Interestingly, NKCC1-null mice exhibit deafness at birth (Delpire et al., 1999). NKCC1 suppression can cause both reversible and irreversible hearing loss (Watabe et al., 2017). Transgenic mice generated to selectively manipulate cochlear NKCC1 resulted in reversible hearing loss in the postnatal mice (Watabe et al., 2017). Therefore, systemic BTN delivery for CNS disorders during critical developmental periods would also inhibit the cochlear-specific NKCC1 isoform (Delpire et al., 2009). The ototoxicity reported following BTN interventions in the HIE trial (NCT01434225, 2015) have been discussed taking the above findings into consideration (Pressler et al., 2015; Allegaert et al., 2016). Interestingly, a animal model study that utilized mice with mutations of Slc12a2 found that the associated inner ear dysfunction additionally caused motor hyperactivity via increased levels of pCREB and pERK in the nucleus accumbens (Antoine et al., 2013). These brain circuit effects were independent of loss of NKCC1 in the brain. Acute diuretic effects of BTN could also lead to dyshomeostasis and fluid imbalance in the inner ear, further aggravating ototoxicity, but in a reversible way. NKCC1-null mice exhibit decreased blood pressure (Flagella et al., 1999), intestinal bleeding (Flagella et al., 1999), infertility (Pace et al., 2000), and salivary secretion reduction (Evans et al., 2000; Sun, 2010). These findings indicate NKCC1 function plays a significant role in multiple organs other than the kidney and brain.

## BTN’s Brain Availability

Prenatal brains have been thought to be more vulnerable to drugs, toxins and pathological conditions due to an immature BBB (Saunders et al., 2012). However, the prevalence of efflux transporters present in the placenta may provide protection *in utero*. This protection is lost after birth and may cause the neonatal period to be more vulnerable than the fetal period (Saunders et al., 2012). However, this understanding has been challenged, especially with regard to neurotoxicology (Ek et al., 2012; Saunders et al., 2012). Trypan blue and other acidic dyes administered systemically have been utilized to investigate the integrity of the BBB (Saunders et al., 2012). Functionally effective tight junctions are present in the embryonic brain (Nitta et al., 2003; Ek et al., 2006; Saunders et al., 2012). Therefore, the neonatal BBB is present and functional during development.

The OAT efflux transporter family is responsible for efficacious drug transport (Kusuhara et al., 1999; Urquhart and Kim, 2009; Nigam et al., 2015). OAT3 mediates the necessary uptake for BTN to reach NKCC in the kidney (Burckhardt, 2012). Probenecid, while an inhibitor of all members of the OAT family, is a more selective inhibitor of OAT3, and was utilized to study the effects of the PKs of BTN in the brain (Donovan et al., 2014, 2016). Probenecid increased the brain levels of BTN (Donovan et al., 2014; Töllner et al., 2015). Therefore, activity of OAT3 may contribute significantly to the poor brain access of BTN after systemic administration (Löscher et al., 2013) (Figure 1B1). Both restricted passive diffusion and active efflux transport by OAT3, murine OAT polypeptide (Oatp1a4), and multidrug resistance protein 4 (MRP4) lowered the concentrations of systemically administered BTN to the brain in *in vivo* experiments (Römermann et al., 2017). While initially thought that OAT3 is the only transporter that actively effluxes BTN, later studies confirmed that additional transporters may be involved (Römermann et al., 2017). Excitotoxic insults, however, could result in the failure of the BBB via glutamatergic actions on NMDA receptors expressed on endothelial cells lining the CNS vasculature (Xhima et al., 2016). This could have potential implications on both the influx and efflux kinetics of BTN in conditions where this is known to occur.

Just as low brain concentrations and rapid CNS efflux of BTN leads to low plasma/brain ratios for BTN; only unbound and non-ionized forms of BTN are able to diffuse across membranes to begin with (Figure 1B2). Based on the calculated pKa of BTN, >99% is ionized at the plasma pH of 7.4 when assessed with nuclear magnetic resonance (NMR) spectroscopy and ultraviolet visible (UV) spectroscopy (Song et al., 2011), with additional variations if using pooled human blood (Walker et al., 1989) or *in vitro* bovine albumin (Donovan et al., 2016) to test binding. Free and unionized BBB-permeable BTN is much lower after systemic administration, compared to what would be warranted for efficacious brain penetration and NKCC1 antagonization in neurons (Puskarjov et al., 2014). Less than 1% of the IP administered BTN (0.15–0.3mg/kg) reached the brain in hypoxic-ischemic insulted P10 rats, showing a similar penetration to those who didn’t receive the insult (Puskarjov et al., 2014) (Figure 1B4). These results indicate that even ischemic injury to the P10 BBB did not help BTN penetrate the brain at higher concentrations.

Overall, systemic IP injections of BTN yield lower levels of free BTN than IV infusions or injections (Olsen, 1977; Brandt et al., 2010; Puskarjov et al., 2014; Wang et al., 2015). Once in plasma, only BTN that is not bound by albumin can enter the brain, skewing the ratio of BTN presence in the brain compared to plasma. Since the concentration of BTN detected in the brain after systemic administration was much lower than what would be needed to antagonize neuronal NKCC1, higher doses of BTN would have to be administered. Doing so, however, would also aggravate all the other systemic or non-neuronal effects of BTN (Löscher et al., 2013). A short half-life and the presence of efflux transporters further challenges the maintenance of BTN levels in the brain. All of these factors indicate poor CNS interaction of BTN from a therapeutic efficacy standpoint. However, BTN, while only approved as a diuretic, has been reported to show beneficial effects in some neurological disorders. Systemic effects due to non-neuronal action on NKCC1 expressed inside and outside of the BBB needs consideration.

## Systemic Effects

Low doses of BTN (0.5–2 mg in adults, 0.1–0.3 mg/kg in neonates and children) are sufficient to induce diuresis. With the above concentrations, diuresis is complete in about 4h’s (FDA Bumetanide Label, 2009). The elimination of BTN is considerably slower in neonatal patients compared with adults (FDA Bumetanide Label, 2009), with ranges from 8 to 27 h in neonates and 33–100 min in adults (Pacifici, 2012; Puskarjov et al., 2014). BTN is also utilized in the treatment of nephrotic syndrome and massive edema (2–6 mg/day) (Lemieux et al., 1981), heart failure (1–3 mg/day) (Kourouklis et al., 1976), and liver disease (0.5–4 mg/day) (Moult et al., 1974). BTN can be administered orally, intravenously, or intramuscularly and increases urinary output by inhibiting Na^+^ and Cl^−^ in the loop of Henle (Figure 1B3) with secondary actions on the proximal tubules (Bourke et al., 1973; Murdoch and Auld, 1975; Ward and Heel, 1984; FDA Bumetanide Label, 2009). Side effects of the induced diuresis include volume depletion, electrolyte depletion, and hypokalemia (FDA Bumetanide Label, 2009). Repeated doses require caution and should not exceed 10 mg a day (FDA Bumetanide Label, 2009).

### Off-Label Studies

#### Neonatal Seizures

The anti-seizure efficacy of BTN by itself or as an adjunct has been evaluated in several pre-clinical models of neonatal seizures (Dzhala et al., 2005, 2008; Cleary et al., 2013; Kang et al., 2015; Kharod et al., 2018). BTN alone hyperpolarized the equilibrium potential of Cl^−^ in immature neurons, suppressed epileptiform activity in hippocampal slices *in vitro* and reduced kainic acid induced seizures *in vivo* (Dzhala et al., 2005). BTN seizure suppression data reported in this study resulted in the initiation of clinical trials for BTN for HIE seizures. This was attributed to the hypothesized higher expression of NKCC1 in immature human and rodent brains (see Figure 1A human and animal WB data). However, the developmentally regulated low expression and function of KCC2 at birth may also play a significant role in determining Cl^−^ gradients during early postnatal weeks (Rivera et al., 1999; Lee et al., 2005). Additionally, microarray data show that human NKCC1 mRNA increases into adulthood, and therefore contradicts the developmental hypothesis of high NKCC1 transporter function and its association with early life seizure susceptibility.

*In vitro*, BTN served as an efficacious adjunct to PB to decrease recurrent tonic-clonic epileptiform activity after application of Mg^2+^ free ACSF in the intact immature hippocampus (Dzhala et al., 2008). PB and BTN applied in combination to *ex vivo* hippocampal slices following hypoxia-induced seizures reversed seizure-induced changes in E_GABA_ when compared to PB and/or BTN applied alone (Cleary et al., 2013). In a *in vivo* model of ischemic seizures, BTN failed as an adjunct to PB in P10 CD-1 mice (Kang et al., 2015). The implications in utilizing *in vivo* models boils down to the simplification that many other factors are at play, often times, these attributes are ones that cannot be controlled as in *in vitro* model counterparts. Post-ischemic P7 CD-1 brains were significantly less susceptible to necrotic infarct injury compared to P10 and P12 for the same insult, with no signs of stroke infarcts detected at P7 in the hypoxic-ischemic model (Kang et al., 2015). While P10 and P12 CD-1 pups responded to PB, P7 pups did not respond to the same loading dose (Kang et al., 2015). PB-inefficacy at P7 was not rescued with co-administration of BTN when administered 1 h post-PB and PB efficacy witnessed in the P10 age group was shunted when BTN was administered, meaning there was significant increase in seizure burden after effective seizure suppression with PB (Kang et al., 2015). In a recent model of pentylenetetrazole (PTZ)-induced acute episodic seizures at the same age (P7) and same mouse strain (CD-1), PB effectively suppressed an even higher seizure burden than what was witnessed in the ischemic seizure model where PB was inefficacious (Kang et al., 2015; Kharod et al., 2018). BTN administration 1h post-PB reversed PB efficacy (Kharod et al., 2018), similar to the ischemic model at P10 (Kang et al., 2015). In contrast, BTN significantly reduced PTZ induced seizure susceptibility following hypoxic-ischemic injury at P7 in a rat model (Hu et al., 2017). The 3-day BTN treatment also helped restore hippocampal neurogenesis and improved cognitive function in the treated rats (Hu et al., 2017). These improvements may suggest the long-term benefits of acute BTN intervention unrelated to the acute modulation of neuronal Cl^−^.

Cl^−^ co-transporter expression levels, following seizure induction, not only differ by type of insults used to induce neonatal seizures in models of pre-clinical research but also by temporal changes from time of the insult (Cleary et al., 2013; Puskarjov et al., 2014; Kharod et al., 2018). In the CD-1 mouse ischemia model, there was a downregulation of KCC2 total protein, while a PTZ insult in the same strain at the same age resulted in a upregulation of KCC2 (Kang et al., 2015; Kharod et al., 2018). No significant changes were detected in NKCC1 expression in either model, indicating KCC2 may play a critical role in acute post-insult brain plasticity in acquired models of seizures. In the ischemia model of neonatal seizures, it is possible the reported BTN-induced aggravation of PB-suppressed seizures was due to PB-rebound seizures. If BTN aggravated the seizures independently, the mechanism is not understood at this point. Furthermore, it is of interest that BTN aggravated PB-suppressed seizures both in the ischemic and PTZ induced models in a sex-specific manner (see Table 1), highlighting the importance of testing sex as a biological variable in every pre-clinical study. Table 1 highlights the bias toward using only male rodents for pre-clinical studies. In summary, BTN has been reported to have varying efficacies in animal models of neonatal seizures (Dzhala et al., 2008; Mares, 2009; Mazarati et al., 2009; Khirug et al., 2010; Cleary et al., 2013; Kang et al., 2015; Kharod et al., 2018) (see Figure 1C and Table 1). These model-specific efficacies of BTN could be explained by multiple factors including but not limited to: (1) post-insult response of Cl^−^ cotransporter expression (both KCC2 and NKCC1), (2) presence of cell-death, edema, albumin leak through disrupted BBB, (3) maturity of the of the BBB (Kang and Kadam, 2014) and (4) the role of non-neuronal cells like astrocytes at tight junctions. Developmentally high NKCC1 expression proposed to result in high [Cl-]_i_ and thus depolarizing GABA was the hypothesis that formed the basis for the clinical trial for BTN intervention in HIE neonates, but BTN failed as an anti-seizure adjunct to PB (NCT01434225, 2015; Pressler et al., 2015).

#### Focal Cortical Dysplasia

Focal cortical dysplasia is a malformation of cortical development (Kabat and Król, 2012). The histological characteristics were first described by Taylor et al. (1971). Three types of cortical dysplasia are recognized (Blümcke et al., 2011), types I, II, and III. Characterization of Type I to Type III FCD is based on the location and extent of histopathological changes associated with cortical dysplasias. In the case of Type III FCD, the dysplasia extends beyond the temporal lobe and is associated with other principal lesions like hippocampal sclerosis or vascular malformations (Blümcke et al., 2011; Kabat and Król, 2012). Dysregulated GABAergic transmissions, either due to disrupted chloride cotransporter function or altered GABA_A_R mediation are some of the reported characteristics of FCD (Blauwblomme et al., 2018). The effect of BTN was assessed in slices from tissue resected from FCD patients. Bath–applied BTN suppressed interictal discharges, in slices from resected tissue which resumed after washout in physiological artificial CSF (Blauwblomme et al., 2018). In this study, the effects of BTN were variable. However, it suppressed interictal discharges in 9 of 12 slices (see Table 1). The conclusions tied the BTN effects on suppression of the interictal discharges to a hypothesized upregulation of NKCC1, without quantification of NKCC1. The results, however, did report reduced membrane KCC2 expression in ictogenic zones within the resected FCD brain slices.

#### Temporal Lobe Epilepsy

Spontaneous rhythmic activity has been reported in brain slices derived from patients with TLE, that were suppressed by glutamatergic or GABAergic signaling antagonists (Cohen et al., 2002). Brain tissue resected from TLE patients showed alterations in the relative expression of KCC2 and NKCC1 in neurons, which may contribute to epileptiform activity in the subiculum of patients with hippocampal sclerosis (Munoz et al., 2007). BTN attenuated seizure frequency in two out of three patients with TLE (Eftekhari et al., 2013). Seizure models for TLE, including amygdala-kindled rats, pilocarpine-induced SE, post-traumatic seizures, neuronal hyperactivity, ischemia-induced seizures and febrile seizures have been utilized to study altered chloride cotransporter levels (Hochman and Schwartzkroin, 2000; Okabe et al., 2002; Yamada et al., 2004; Li et al., 2008; Lee et al., 2011; Koyama et al., 2012; Kaila et al., 2014; Sivakumaran and Maguire, 2016). The excitatory GABA caused by NKCC1 upregulation remains the proposed rationale behind testing BTN’s efficacy (Ben-Ari, 2017). The contributing role of KCC2 hypofunction in seizure susceptibilities is also being explored (Chen et al., 2017). In a recent study, it was shown that local ablation of KCC2 activity in a subset of hippocampal neurons resulted in compromised GABAergic inhibition and development of spontaneous seizures and hippocampal sclerosis (Kelley et al., 2018).

#### Autism

Autism and prevalence of seizures go hand-in-hand (Besag, 2017). The seizures in patients with autism are often treatment-resistant (Sansa et al., 2011). High [Cl^−^]_i_ makes GABA excitatory, and was proposed to be the basis of the contradictory actions of PB in autistic patients with seizures (Lemonnier et al., 2012). Based on these hypotheses, reducing the [Cl^−^]_i_ via BTN proved efficacious in an animal model of autism using valproic acid exposure (Tyzio et al., 2014). In a commentary response to this study, however, it was noted that it is premature to consider BTN as a prenatal intervention suitable for ASD due to the lack of proper technical tests and failures to assess the long lasting modifications (Bambini-Junior et al., 2014).

In three separate clinical trials where BTN was administered to patients with autism ranging from infancy to adulthood, BTN significantly improved Childhood Autistic Rating Scale (CARS) scores and attenuated the severity of the disorder overall, with no major side effects other than diuresis (Lemonnier and Ben-Ari, 2010; Ben-Ari, 2017; Lemonnier et al., 2017). In a recent study, BTN given to a subset of the patients with autism showed the normalization of amygdala activation upon eye contact (Hadjikhani et al., 2018) (Figure 1C), long-after cessation of the BTN therapy suggesting permanent and corrective alterations to the underlying circuits.

#### Schizophrenia

Increased NKCC1 mRNA expression in patients with schizophrenia was also the proposed rationale underlying BTN treatment trials in these patients. Many patients with schizophrenia manifest clinical symptoms that suggest prefrontal cortex dysfunction (Weinberger, 1988), and so this region remains of interest to study under pathological conditions. A 7.4-fold upregulation of NKCC1 mRNA was detected in the Brodmann’s area 46 in schizophrenia patients (Dean et al., 2007). However, more recent data elucidate DLPFC NKCC1b mRNA was significantly decreased in patients with schizophrenia and NKCC1a mRNA remained unchanged when compared to controls (Morita et al., 2014). This finding indicates that NKCC1 isoform expression underlying different pathological conditions could differ by the neurological disorder. In both a case study (Lemonnier et al., 2016) and a small pilot study (Rahmanzadeh et al., 2017), BTN reduced hallucinations in schizophrenic patients. In additional tests, however, where the brief psychiatric rating scale (BPRS) was assessed, BTN treatment had no significant effect when compared to the placebo group (Rahmanzadeh et al., 2016).

In schizophrenia and autism, and in the cases where increased NKCC1 expression has been determined, either by western blotting or PCR, it would be of interest to investigate whether the developmental profile of NKCC1 expression is impaired. The potential developmental and functional alterations in NKCC1 isoform expression and distribution both in healthy and diseased brains could help understand the role of NKCC1 in CNS disorders.

## Shared Mechanisms With Osmotic Agents

Osmotic agents have been administered for treatment of seizures and alleviation of brain injury and edema (Cruz et al., 2004; Maa et al., 2011; Walcott et al., 2012). Osmotic agents may share mechanism of action(s) with BTN due to their shared diuretic properties. Mannitol (an osmolyte), much like BTN, has been reported to have varying efficacies. The anti-seizure effects of mannitol have long been studied in animal models and humans. In a kainic acid rat model, mannitol (1.5 g/kg, IV, 10 min, 1.5 and 3 h, respectively, after kainic acid administration) yielded a protective effect at 1.5 h after kainic acid seizure induction (Baran et al., 1987). Along with anti-seizure effects, mannitol prevented the formations of lesions and other potential neurochemical changes. Additionally, rat CA1 hippocampal slices in solutions made 5–30 mosmol/kg hyperosmotic by additions of mannitol, sucrose, raffinose, L-glucose and dextran blocked [K+]o-induced spontaneous seizures (Traynelis and Dingledine, 1989). In contrast, a recent study did not find any anticonvulsant effects of NKCC blockers (LD’s) in a electroconvulsive adult seizure model, while other diuretics exhibited some activity at high doses (Załuska et al., 2018). Haglund and Hochman (2005) administered a single dose of either 20 mg furosemide (cation-Cl^−^ cotransporter antagonist) or 50 g mannitol to epileptic patients during their surgical procedures for the treatment of intractable epilepsy; both drugs significantly suppressed epileptic-spikes and electrical stimulation-evoked epileptiform discharges in all subjects recorded from electrodes directly placed on the cortical surface. In another study, mannitol was given to pediatric patients experiencing status epilepticus (SE) and raised intracranial pressure. While success rates were not provided in detail, authors concluded that all seizures cannot be treated with one drug. Underlying pathologies must be taken into consideration when choosing what anti-seizure drug to employ, especially in regards to the utilization of diuretics, which have preexisting conflicting success rates (Smith et al., 1996). One prominent mechanism shared by diuretics in general, with proposed antiepileptic efficacy is inhibition of carbonic anhydrase. Carbonic anhydrases catalyze reversible hydration/dehydration of CO_2_/HCO_3_^−^, respectively (Aggarwal et al., 2013). These actions suppress seizures through disruption of CO_2_ equilibrium with inhibitory action on ion channels (Aggarwal et al., 2013). However, carbonic anhydrase inhibition likely is not one of the mechanisms of action of BTN, since BTN is a weak carbonic anhydrase inhibitor (isoforms I, II, III, and XIII) (Carta and Supuran, 2013). BTN was inhibitory to carbonic anhydrase for tumor-associated isoforms (Carta and Supuran, 2013). Therefore, BTN’s mechanism of action via carbonic anhydrase requires further investigations to fully understand its anti-convulsive properties.

## Btn Pro-Drugs and Analogs

To improve BTN accessibility to the brain, pro-drugs with lipophilic and uncharged esters, alcohol and amide analogs have been created. These pro-drugs convert to BTN after gaining access into the brain. There was a significantly higher concentration of ester prodrug, BUM5 (*N*,*N* – dimethylaminoethyl ester), in mouse brains compared to the parent BTN (10 mg/kg, IV of BTN and equimolar dose of 13 mg/kg, IV of BUM5) (Töllner et al., 2014). BUM5 stopped seizures in adult animal models where BTN failed to work (Töllner et al., 2014; Erker et al., 2016). BUM5 was also less diuretic and showed better brain access when compared to the other prodrugs, BUM1 (ester prodrug), BUM7 (alcohol prodrug) and BUM10 (amide prodrug). BUM5 was reported to be more effective than BTN in altering seizure thresholds in epileptic animals post-SE and post-kindling (Töllner et al., 2014). Furthermore, BUM5 (13 mg/kg, IV) was more efficacious than BTN (10 mg/kg, IV) in promoting the anti-seizure effects of PB, in a maximal electroshock seizure model (Erker et al., 2016). Compared to BUM5 which was an efficacious adjunct to PB in the above mentioned study, BTN was not efficacious when administered as an adjunct (Erker et al., 2016). In addition to seizure thresholds, further studies need to be conducted to assess effects of BUM5 on seizure burdens, ictal events, duration and latencies.

Recently, a benzylamine derivative, bumepamine, has been investigated in pre-clinical models. Since benzylamine derivatives lack the carboxylic group of BTN, it results in lower diuretic activity (Nielsen and Feit, 1978). This prompted Brandt et al. (2018) to explore the proposed lower diuretic activity, higher lipophilicity and lower ionization rate of bumepamine at physiological pH. Since it is known that rodents metabolize BTN quicker than humans, the study used higher doses of 10 mg/kg of bumepamine similar to their previous BTN studies (Olsen, 1977; Brandt et al., 2010; Töllner et al., 2014). Bumepamine, while only being nominally metabolized to BTN, was more effective than BTN to support anticonvulsant effects of PB in rodent models of epilepsy. This GABAergic response, however, was not due to antagonistic actions on NKCC1; suggesting bumepamine may have an off-target effect, which remains unknown. However, the anticonvulsive effects of bumepamine, in spite of its lack of action on NKCC1, are to be noted. Additionally, in another study by the same group, it was shown that azosemide was 4-times more potent an inhibitor of NKCC1 than BTN, opening additional avenues for better BBB penetration and NKCC1-antagonizing compounds for potential neurological drug discovery (Hampel et al., 2018).

## Conclusion

The beneficial effects of BTN reported in cases of autism, schizophrenia and TLE, given its poor-brain bioavailability are intriguing. The mechanisms underlying the effects of BTN, as a neuromodulator for developmental and neuropsychiatric disorders could be multifactorial due to prominent NKCC1 function at neuronal and non-neuronal sites within the CNS. Investigation of the possible off-target and systemic effects of BTN may help further this understanding with the advent of a new generation of brain-accessible BTN analogs.

## Author Contributions

SCK, SKK, and SDK contributed to writing of this manuscript. SDK supervised and made final edits.

## Conflict of Interest Statement

The authors declare that the research was conducted in the absence of any commercial or financial relationships that could be construed as a potential conflict of interest.

## Footnotes

1 http://braincloud.jhmi.edu/

## Abbreviations

BBB: blood–brain barrier
BTN: bumetanide
Cl^−^: chloride
CNS: central nervous system
DLPFC: dorsolateral prefrontal cortex
FCD: focal cortical dysplasia
GABA: gamma-aminobutyric acid
HIE: hypoxic-ischemic encephalopathy
IP: intraperitoneal
IV: intravenous
KCC2: K-Cl cotransporter 2
LD: loop diuretic
NKCC: Na-K-Cl cotransporter
OAT: organic anion transporter
PB: phenobarbital
PD: pharmacodynamics
PK: pharmacokinetic
TLE: temporal lobe epilepsy.

## References

[B1] Abbas L. Whitfield T. T. (2009). Nkcc1 (Slc12a2) is required for the regulation of endolymph volume in the otic vesicle and swim bladder volume in the zebrafish larva. *Development* 136 2837–2848. 10.1242/dev.034215 19633174 PMC2730410

[B2] Aggarwal M. Kondeti B. McKenna R. (2013). Anticonvulsant/antiepileptic carbonic anhydrase inhibitors: a patent review. *Expert Opin. Ther. Pat.* 23 717–724. 10.1517/13543776.2013.782394 23514045

[B3] Allegaert K. Lahav A. Van den Anker J. N. (2016). A mechanism to explain ototoxicity in neonates exposed to bumetamide: lessons to help improve future product development in neonates. *Pediatr. Drugs* 18 331–333. 10.1007/s40272-016-0190-4 27538744

[B4] Amin H. Marinaro F. De Pietri Tonelli D. Berdondini L. (2017). Developmental excitatory-to-inhibitory GABA-polarity switch is disrupted in 22q11.2 deletion syndrome: a potential target for clinical therapeutics. *Sci. Rep.* 7:15752. 10.1038/s41598-017-15793-9 29146941 PMC5691208

[B5] Annunziato L. Boscia F. Pignataro G. (2013). Ionic transporter activity in astrocytes, microglia, and oligodendrocytes during brain ischemia. *J. Cereb. Blood Flow Metab.* 33 969–982. 10.1038/jcbfm.2013.44 23549380 PMC3705429

[B6] Antoine M. W. Hübner C. A. Arezzo J. C. Hébert J. M. (2013). A causative link between inner ear defects and long-term striatal dysfunction. *Science* 341 1120–1123. 10.1126/science.1240405 24009395 PMC4731229

[B7] Antrobus S. P. Lytle C. Payne J. A. (2012). K+-Cl- cotransporter-2 KCC2 in chicken cardiomyocytes. *Am. J. Physiol. Cell Physiol.* 303 C1180–C1191. 10.1152/ajpcell.00274.2012 23034386 PMC3530769

[B8] Asbury M. J. Gatenby P. B. O’Sullivan S. Bourke E. (1972). Bumetanide: potent new ‘Loop’ diuretic. *Br. Med. J.* 1 211–213. 10.1136/bmj.1.5794.211 4550872 PMC1789187

[B9] Bambini-Junior V. Nunes G. D. Schneider T. Gottfried C. (2014). Comment on ‘oxytocin-mediated gaba inhibition during delivery attenuates autism pathogenesis in rodent offspring.’ *Science* 346:176. 10.1126/science.1255679 25301610

[B10] Baran H. Lassmann H. Sperk G. Seitelberger F. Hornykiewicz O. (1987). Effect of mannitol treatment on brain neurotransmitter markers in kainic acid-induced epilepsy. *Neuroscience* 21 679–684. 10.1016/0306-4522(87)90029-7 3114666

[B11] Ben-Ari Y. (2017). NKCC1 chloride importer antagonists attenuate many neurological and psychiatric disorders. *Trends Neurosci.* 40 536–554. 10.1016/j.tins.2017.07.001 28818303

[B12] Ben-Ari Y. Khalilov I. Kahle K. T. Cherubini E. (2012). The GABA excitatory/inhibitory shift in brain maturation and neurological disorders. *Neuroscientist* 18 467–486. 10.1177/1073858412438697 22547529

[B13] Besag F. M. (2017). Epilepsy in patients with autism: links, risks and treatment challenges. *Neuropsychiatr. Dis. Treat.* 14 1–10. 10.2147/NDT.S120509 29296085 PMC5739118

[B14] Blauwblomme T. Dossi E. Pellegrino C. Goubert E. Iglesias B. G. Sainte-Rose C. (2018). Gamma-aminobutyric acidergic transmission underlies interictal epileptogenicity in pediatric focal cortical dysplasia. *Ann. Neurol.* 85 204–217. 10.1002/ana.25403 30597612

[B15] Blümcke I. Thom M. Aronica E. Armstrong D. D. Vinters H. V. Palmini A. (2011). The clinicopathologic spectrum of focal cortical dysplasias: a consensus classification proposed by an ad hoc task force of the ILAE diagnostic methods commission. *Epilepsia* 52 158–174. 10.1111/j.1528-1167.2010.02777.x 21219302 PMC3058866

[B16] Bourke E. Asbury M. J. O’Sullivan S. Gatenby P. B. (1973). The sites of action of bumetanide in man. *Eur. J. Pharmacol.* 23 283–289. 10.1016/0014-2999(73)90096-4 4583564

[B17] Bragin D. E. Sanderson J. L. Peterson S. Connor J. A. Müller W. S. (2009). Development of epileptiform excitability in the deep entorhinal cortex after status epilepticus. *Eur. J. Neurosci.* 30 611–624. 10.1111/j.1460-9568.2009.06863.x 19674083 PMC2776653

[B18] Brandt C. Nozadze M. Heuchert N. Rattka M. Löscher W. (2010). Disease-modifying effects of phenobarbital and the NKCC1 inhibitor bumetanide in the pilocarpine model of temporal lobe epilepsy. *J. Neurosci.* 30 8602–8612. 10.1523/JNEUROSCI.0633-10.2010 20573906 PMC6634618

[B19] Brandt C. Seja P. Töllner K. Römermann K. Hampel P. Kalesse M. (2018). Bumepamine, a brain-permeant benzylamine derivative of bumetanide, does not inhibit NKCC1 but is more potent to enhance phenobarbital’s anti-seizure efficacy. *Neuropharmacology* 143 186–204. 10.1016/j.neuropharm.2018.09.025 30248303

[B20] Brater C. D. (2000). Pharmacology of diuretics. *Am. J. Med. Sci.* 319 38–50. 10.1016/S0002-9629(15)40678-0 10653443

[B21] Burckhardt G. (2012). Drug transport by organic anion transporters (OATs). *Pharmacol. Ther.* 136 106–130. 10.1016/j.pharmthera.2012.07.010 22841915

[B22] Carta F. Supuran C. T. (2013). Diuretics with carbonic anhydrase inhibitory action: a patent and literature review (2005 – 2013). *Expert Opin. Ther. Pat.* 23 681–691. 10.1517/13543776.2013.780598 23488823

[B23] Chen L. Wan L. Wu Z. Ren W. Huang Y. Qian B. (2017). KCC2 downregulation facilitates epileptic seizures. *Sci. Rep.* 7:156. 10.1038/s41598-017-00196-7 28279020 PMC5427808

[B24] Clayton G. H. Owens G. C. Wolff J. S. Smith R. L. (1998). Ontogeny of cation-Cl- cotransporter expression in rat neocortex. *Brain Res. Dev. Brain Res.* 109 281–292. 10.1016/S0165-3806(98)00078-9 9729431

[B25] Cleary R. T. Sun H. Huynh T. Manning S. M. Li Y. Rotenberg A. (2013). Bumetanide enhances phenobarbital efficacy in a rat model of hypoxic neonatal seizures. *PLoS One* 8:e57148. 10.1371/journal.pone.0057148 23536761 PMC3594228

[B26] Cohen I. Navarro V. Clemenceau S. Baulac M. Miles R. (2002). On the origin of interictal activity in human temporal lobe epilepsy in vitro. *Science* 298 1418–1421. 10.1126/science.1076510 12434059

[B27] Colantuoni C. Lipska B. K. Ye T. Hyde T. M. Tao R. Leek J. T. (2011). Temporal dynamics and genetic control of transcription in the human prefrontal cortex. *Nature* 478 519–523. 10.1038/nature10524 Available at: http://braincloud.jhmi.edu/ for Brain Cloud Expression data information. 22031444 PMC3510670

[B28] Côme E. Heubl M. Schwartz E. J. Poncer J. C. Lévi S. (2019). Reciprocal regulation of KCC2 trafficking and synaptic activity. *Front. Cell. Neurosci.* 13:48. 10.3389/fncel.2019.00048 30842727 PMC6391895

[B29] Conti L. Palma E. Roseti C. Lauro C. Cipriani R. de Groot M. (2011). Anomalous levels of Cl- transporters cause a decrease of GABAergic inhibition in human peritumoral epileptic cortex. *Epilepsia* 52 1635–1644. 10.1111/j.1528-1167.2011.03111.x 21635237

[B30] Crouch J. J. Sakaguchi N. Lytle C. Schulte B. A. (1997). Immunohistochemical localization of the Na-K-Cl Co-transporter (NKCC1) in the gerbil inner ear. *J. Histochem. Cytochem.* 45 773–778. 10.1177/002215549704500601 9199662

[B31] Cruz J. Minoja G. Okuchi K. Facco E. (2004). Successful use of the new high-dose mannitol treatment in patients with Glasgow coma scale scores of 3 and bilateral abnormal pupillary widening: a randomized trial. *J. Neurosurg.* 100 376–383. 10.3171/jns.2004.100.3.0376 15035271

[B32] Damier P. Hammond C. Ben-Ari Y. (2016). Bumetanide to treat Parkinson disease: a report of 4 cases. *Clin. Neuropharmacol.* 39 57–59. 10.1097/WNF.0000000000000114 26757306

[B33] Dean B. Keriakous D. Scarr E. Thomas E. A. (2007). Gene expression profiling in brodmann’s area 46 from subjects with schizophrenia. *Aust. N. Z. J. Psychiatry* 41 308–320. 10.1080/00048670701213245 17464717

[B34] Deidda G. Parrini M. Naskar S. Bozarth I. F. Contestabile A. Cancedda L. (2015). Reversing excitatory GABAAR signaling restores synaptic plasticity and memory in a mouse model of down syndrome. *Nat. Med.* 21 318–326. 10.1038/nm.3827 25774849

[B35] Delpire E. Days E. Lewis L. M. Mi D. Kim K. Lindsley C. W. (2009). Small-molecule screen identifies inhibitors of the neuronal K-Cl cotransporter KCC2. *Proc. Natl. Acad. Sci. U.S.A.* 106 5383–5388. 10.1073/pnas.0812756106 19279215 PMC2654392

[B36] Delpire E. Lu J. England R. Dull C. Thorne T. (1999). Deafness and imbalance associated with inactivation of the secretory Na-K-2Cl Co-transporter. *Nat. Genet.* 22 192–195. 10.1038/9713 10369265

[B37] Donovan M. D. O’Brien F. E. Boylan G. B. Cryan J. F. Griffin B. T. (2014). The effect of organic anion transporter 3 inhibitor probenecid on bumetanide levels in the brain: an integrated in vivo microdialysis study in the rat. *J. Pharm. Pharmacol.* 67 501–510. 10.1111/jphp.12341 25490899

[B38] Donovan M. D. Schellekens H. Boylan G. B. Cryan J. F. Griffin B. T. (2016). In vitro bidirectional permeability studies identify pharmacokinetic limitations of NKCC1 inhibitor bumetanide. *Eur. J. Pharmacol.* 770 117–125. 10.1016/j.ejphar.2015.12.001 26673740

[B39] Du L. Shan L. Wang B. Li H. Xu Z. Staal W. G. (2015). A pilot study on the combination of applied behavior analysis and bumetanide treatment for children with autism. *J. Child Adolesc. Psychopharmacol.* 25 585–588. 10.1089/cap.2015.0045 26258842

[B40] Dzhala V. I. Brumback A. C. Staley K. J. (2008). Bumetanide enhances phenobarbital efficacy in a neonatal seizure model. *Ann. Neurol.* 63 222–235. 10.1002/ana.21229 17918265

[B41] Dzhala V. I. Talos D. M. Sdrulla D. A. Brumback A. C. Mathews G. C. Benke T. A. (2005). NKCC1 transporter facilitates seizures in the developing brain. *Nat. Med.* 11 1205–1213. 10.1038/nm1301 16227993

[B42] Eftekhari S. Habibabadi J. M. Ziarani M. N. Fesharaki S. S. H. Gharakhani M. Mostafavi H. (2013). Bumetanide reduces seizure frequency in patients with temporal lobe epilepsy. *Epilepsia* 54 e9–e12. 10.1111/j.1528-1167.2012.03654.x 23061490

[B43] Ek C. J. Dziegielewska K. M. Habgood M. D. Saunders N. R. (2012). Barriers in the developing brain and neurotoxicology. *Neurotoxicology* 33 586–604. 10.1016/j.neuro.2011.12.009 22198708

[B44] Ek C. J. Dziegielewska K. M. Stolp H. Saunders N. R. (2006). Functional effectiveness of the blood-brain barrier to small water soluble molecules in developing and adult opossum (*Monodelphis domestica*). *J. Comp. Neurol.* 496 13–26. 10.1002/cne.20885 16528724 PMC2634607

[B45] Erker T. Brandt C. Töllner K. Schreppel P. Twele F. Schidlitzki A. (2016). The bumetanide prodrug BUM5, but not bumetanide, potentiates the antiseizure effect of phenobarbital in adult epileptic mice. *Epilepsia* 57 698–705. 10.1111/epi.13346 26921222

[B46] Evans R. L. Park K. Turner R. J. Watson G. E. Nguyen H. V. Dennnett M. R. (2000). Severe impairment of salivation in Na+/K+/2Cl- cotransporter (NKCC1)-deficient mice. *J. Biol. Chem.* 275 26720–26726. 10.1074/jbc.M003753200 10831596

[B47] FDA Bumetanide Label (2009). *FDA Label.* Silver Spring, MD: FDA.

[B48] Flagella M. Clarke L. L. Miller M. L. Erway L. C. Giannella R. A. Andringa A. (1999). Mice lacking the basolateral Na-K-2Cl cotransporter have impaired epithelial chloride secretion and are profoundly deaf. *J. Biol. Chem.* 274 26946–26955. 10.1074/jbc.274.38.26946 10480906

[B49] Geck P. Pietrzyk C. Burckhardt B. C. Pfeifferl B. Heinz E. (1980). Electrically silent cotransport of Na+, K+ and Cl- in Ehrlich cells. *Biochim. Biophys. Acta* 600 432–447. 10.1016/0005-2736(80)90446-0 7407122

[B50] Hadjikhani N. Asberg Johnels J. Lassalle A. Zurcher N. R. Hippolyte L. Gillberg C. (2018). Bumetanide for autism: more eye contact, less amygdala activation. *Sci. Rep.* 8:3602. 10.1038/s41598-018-21958-x 29483603 PMC5827728

[B51] Haglund M. M. Hochman D. W. (2005). Furosemide and mannitol suppression of epileptic activity in the human brain. *J. Neurophysiol.* 94 907–918. 10.1152/jn.00944.2004 15728766

[B52] Hampel P. Römermann K. MacAulay N. Löscher W. (2018). Azosemide is more potent than bumetanide and various other loop diuretics to inhibit the sodium-potassium-chloride-cotransporter human variants HNKCC1A and HNKCC1B. *Sci. Rep.* 8:9877. 10.1038/s41598-018-27995-w 29959396 PMC6026185

[B53] Hertz L. (1965). Possible role of neuroglia: a potassium-mediated neuronal – neuroglial – neuronal impulse transmission system. *Nature* 206 1091–1094. 10.1038/2061091a0 5325441

[B54] Hesdorffer D. C. Hauser W. A. Annegers J. F. Rocca W. A. (1996). Severe, uncontrolled hypertension and adult-onset seizures: a case-control study in Rochester, Minnesota. *Epilepsia* 37 736–741. 10.1111/j.1528-1157.1996.tb00644.x 8764811

[B55] Hesdorffer D. C. Stables J. P. Hauser W. A. Annegers J. F. Cascino G. (2001). Are certain diuretics also anticonvulsants? *Ann. Neurol.* 50 458–462. 11601497 10.1002/ana.1136

[B56] Hochman D. W. Schwartzkroin P. A. (2000). Chloride-cotransport blockade desynchronizes neuronal discharge in the ‘Epileptic’ hippocampal slice. *J. Neurophysiol.* 83 406–417. 10.1152/jn.2000.83.1.406 10634883

[B57] Holmes G. L. Tian C. Hernan A. E. Flynn S. Camp D. Barry J. (2015). Alterations in sociability and functional brain connectivity caused by early-life seizures are prevented by bumetanide. *Neurobiol. Dis.* 77 204–219. 10.1016/j.nbd.2015.02.015 25766676 PMC4682568

[B58] Hu J. J. Yang X. L. Luo W. D. Han S. Yin J. Liu W. H. (2017). Bumetanide reduce the seizure susceptibility induced by pentylenetetrazol via inhibition of aberrant hippocampal neurogenesis in neonatal rats after hypoxia-ischemia. *Brain Res. Bull.* 130 188–199. 10.1016/j.brainresbull.2017.01.022 28161194

[B59] Hubner C. A. Stein V. Hermans-Borgmeyer I. Meyer T. Ballanyi K. Jentsch T. J. (2001). Disruption of KCC2 reveals an essential role of K-Cl cotransport already in early synaptic inhibition. *Neuron* 30 515–524. 10.1016/S0896-6273(01)00297-5 11395011

[B60] Hyde T. M. Lipska B. K. Ali T. Mathew S. V. Law A. J. Metitiri O. E. (2011). Expression of GABA signaling molecules KCC2, NKCC1, and GAD1 in cortical development and schizophrenia. *J. Neurosci.* 31 11088–11095. 10.1523/JNEUROSCI.1234-11.2011 21795557 PMC3758549

[B61] Ikeda M. Toyoda H. Yamada J. Okabe A. Sato K. Hotta Y. (2003). Differential development of cation-chloride cotransporters and Cl- homeostasis contributes to differential GABAergic actions between developing rat visual cortex and dorsal lateral geniculate nucleus. *Brain Res.* 984 149–159. 10.1016/S0006-8993(03)03126-3 12932849

[B62] Jantzie L. L. Hu M. Y. Park H. K. Jackson M. C. Yu J. Maxwell J. R. (2015). Chloride cotransporter NKCC1 inhibitor bumetanide protects against white matter injury in a rodent model of periventricular leukomalacia. *Pediatr. Res.* 77 554–562. 10.1038/pr.2015.9 25585037

[B63] Juhn S. K. Ikeda K. Morizono T. Murphy M. (1991). Pathophysiology of inner ear fluid imbalance. *Acta Otolaryngol. Suppl.* 485 9–14. 10.3109/00016489109128038 1843177

[B64] Kabat J. Król P. (2012). Focal cortical dysplasia - review. *Pol. J. Radiol.* 77 35–43. 10.12659/PJR.882968 PMC3403799 22844307

[B65] Kahle K. T. Barnett S. M. Sassower K. C. Staley K. J. (2009). Decreased seizure activity in a human neonate treated with bumetanide, an inhibitor of the Na+-K+-2Cl- cotransporter NKCC1. *J. Child Neurol.* 24 572–576. 10.1177/0883073809333526 19406757

[B66] Kaila K. Price T. J. Payne J. A. Puskarjov M. Voipio J. (2014). Cation-chloride cotransporters in neuronal development, plasticity and disease. *Nat. Rev. Neurosci.* 15 637–654. 10.1038/nrn3819 25234263 PMC4294553

[B67] Kanaka C. Ohno K. Okabe A. Kuriyama K. Itoh T. Fukuda A. (2001). The differential expression patterns of messenger RNAs encoding K-Cl cotransporters (KCC1,2) and Na-K-2Cl cotransporter (NKCC1) in the rat nervous system. *Neuroscience* 104 933–946. 10.1016/S0306-4522(01)00149-X 11457581

[B68] Kang S. K. Kadam S. D. (2014). Pre-clinical models of acquired neonatal seizures: differential effects of injury on function of chloride Co-transporters. *Austin J. Cerebrovasc. Dis. Stroke* 1:1026. 25590049 PMC4290373

[B69] Kang S. K. Markowitz G. J. Kim S. T. Johnston M. V. Kadam S. D. (2015). Age- and sex-dependent susceptibility to phenobarbital-resistant neonatal seizures: role of chloride Co-transporters. *Front. Cell. Neurosci.* 9:173. 10.3389/fncel.2015.00173 26029047 PMC4429249

[B70] Kanner A. M. (2002). Diuretics as antiepileptic drugs. *Epilepsy Curr.* 2 39–40. 10.1046/j.1535-7597.2002.00013.x 15309161 PMC320962

[B71] Kelley M. R. Cardarelli R. A. Smalley J. L. Ollerhead T. A. Andrew P. M. Brandon N. J. (2018). Locally reducing KCC2 activity in the hippocampus is sufficient to induce temporal lobe epilepsy. *EBiomedicine* 32 62–71. 10.1016/j.ebiom.2018.05.029 29884458 PMC6020795

[B72] Kharod S. C. Carter B. M. Kadam S. D. (2018). Pharmaco-resistant neonatal seizures: critical mechanistic insights from a chemoconvulsant model. *Dev. Neurobiol.* 78 1117–1130. 10.1002/dneu.22634 30136373 PMC6214781

[B73] Khirug S. Ahmad F. Puskarjov M. Afzalov R. Kaila K. Blaesse P. (2010). A single seizure episode leads to rapid functional activation of KCC2 in the neonatal rat hippocampus. *J. Neurosci.* 30 12028–12035. 10.1523/JNEUROSCI.3154-10.2010 20826666 PMC6633538

[B74] Kim S. J. Choi J. Y. Son E. J. Namkung W. Lee M. G. Yoon J. H. (2007). Interleukin-1β upregulates Na+-K+-2Cl- cotransporter in human middle ear epithelia. *J. Cell. Biochem.* 101 576–586. 10.1002/jcb.21216 17211836

[B75] Kimelberg H. K. (1992). Astrocytic edema in CNS trauma. *J. Neurotrauma* 9(Suppl. 1), S71–S81. 1588633

[B76] Kourdougli N. Pellegrino C. Renko J. M. Khirug S. Chazal G. Kukko-Lukjanov T. K. (2017). Depolarizing γ-aminobutyric acid contributes to glutamatergic network rewiring in epilepsy. *Ann. Neurol.* 81 251–265. 10.1002/ana.24870 28074534

[B77] Kourouklis C. Christensen O. Augoustakis D. (1976). Bumetanide in congestive heart failure. *Curr. Med. Res. Opin.* 4 422–431. 10.1185/03007997609111998 793779

[B78] Koyama R. Tao K. Sasaki T. Ichikawa J. Miyamoto D. Muramatsu R. (2012). GABAergic excitation after febrile seizures induces ectopic granule cells and adult epilepsy. *Nat. Med.* 18 1271–1278. 10.1038/nm.2850 22797810

[B79] Kusuhara H. Sekine T. Utsunomiya-Tate N. Tsuda M. Kojima R. Cha S. H. (1999). Molecular cloning and characterization of a new multispecific organic anion transporter from rat brain. *J. Biol. Chem.* 274 13675–13680. 10.1074/jbc.274.19.13675 10224140

[B80] Lee H. Chen C. X. Liu Y. J. Aizenman E. Kandler K. (2005). KCC2 expression in immature rat cortical neurons is sufficient to switch the polarity of GABA responses. *Eur. J. Neurosci.* 21 2593–2599. 10.1111/j.1460-9568.2005.04084.x 15932617 PMC2945502

[B81] Lee H. A. Hong S. H. Kim J. W. Jang I. S. (2010). Possible involvement of DNA methylation in NKCC1 gene expression during postnatal development and in response to ischemia. *J. Neurochem.* 114 520–529. 10.1111/j.1471-4159.2010.06772.x 20456012

[B82] Lee H. H. Deeb T. Z. Walker J. A. Davies P. A. Moss S. J. (2011). NMDA receptor activity downregulates KCC2 resulting in depolarizing GABA(A) receptor mediated currents. *Nat. Neurosci.* 14 736–743. 10.1038/nn.2806 21532577 PMC3102766

[B83] Lemieux G. Beauchemin M. Gougoux A. Vinay P. (1981). Treatment of nephrotic edema with bumetanide. *Can. Med. Assoc. J.* 125 1111–1112. 7034916 PMC1862648

[B84] Lemonnier E. Ben-Ari Y. (2010). The diuretic bumetanide decreases autistic behaviour in five infants treated during 3 months with no side effects. *Acta Paediatr.* 99 1885–1888. 10.1111/j.1651-2227.2010.01933.x 20608900

[B85] Lemonnier E. Degrez C. Phelep M. Tyzio R. Josse F. Grandgeorge M. (2012). A randomised controlled trial of bumetanide in the treatment of autism in children. *Transl. Psychiatry* 2:e202. 10.1038/tp.2012.124 23233021 PMC3565189

[B86] Lemonnier E. Lazartigues A. Ben-Ari Y. (2016). Treating schizophrenia with the diuretic bumetanide: a case report. *Clin. Neuropharmacol.* 39 115–117. 10.1097/WNF.0000000000000136 26966887

[B87] Lemonnier E. Villeneuve N. Sonie S. Serret S. Rosier A. Roue M. (2017). Effects of bumetanide on neurobehavioral function in children and adolescents with autism spectrum disorders. *Transl. Psychiatry* 7:e1056. 10.1038/tp.2017.10 28291262 PMC5416661

[B88] Lenart B. Kintner D. B. Shull G. E. Sun D. (2004). Na-K-Cl cotransporter-mediated intracellular Na+ accumulation affects Ca2+ signaling in astrocytes in an in vitro ischemic model. *J. Neurosci.* 24 9585–9597. 10.1523/JNEUROSCI.2569-04.2004 15509746 PMC6730155

[B89] Li H. Tornberg J. Kaila K. Airaksinen M. S. Rivera C. (2002). Patterns of cation-chloride cotransporter expression during embryonic rodent CNS development. *Eur. J. Neurosci.* 16 2358–2370. 10.1046/j.1460-9568.2002.02419.x 12492431

[B90] Li X. Zhou J. Chen Z. Chen S. Zhu F. Zhou L. (2008). Long-term expressional changes of Na+ - K+ - Cl- Co-transporter 1 (NKCC1) and K+ - Cl- Co-transporter 2 (KCC2) in CA1 region of hippocampus following lithium-pilocarpine induced status epilepticus (PISE). *Brain Res.* 1221 141–146. 10.1016/j.brainres.2008.04.047 18550034

[B91] Liu Y. Shangguan Y. Barks J. D. Silverstein F. S. (2012). Bumetanide augments the neuroprotective efficacy of phenobarbital plus hypothermia in a neonatal hypoxia-ischemia model. *Pediatr. Res.* 71 559–565. 10.1038/pr.2012.7 22398701 PMC4721236

[B92] Löscher W. Puskarjov M. Kaila K. (2013). Cation-chloride cotransporters NKCC1 and KCC2 as potential targets for novel antiepileptic and antiepileptogenic treatments. *Neuropharmacology* 69 62–74. 10.1016/j.neuropharm.2012.05.045 22705273

[B93] Lu K. T. Wu C. Y. Cheng N. C. Wo Y. Y. Yang J. T. Yen H. H. (2006). Inhibition of the Na+–K+–2Cl–cotransporter in choroid plexus attenuates traumatic brain injury-induced brain edema and neuronal damage. *Eur. J. Pharmacol.* 548 99–105. 10.1016/j.ejphar.2006.07.048 16962576

[B94] Lu K. T. Wu C. Y. Yen H. H. Peng J. H. Wang C. L. Yang Y. L. (2007). Bumetanide administration attenuated traumatic brain injury through IL-1 overexpression. *Neurol. Res.* 29 404–409. 10.1179/016164107X204738 17626737

[B95] Maa E. H. Kahle K. T. Walcott B. P. Spitz M. C. Staley K. J. (2011). Diuretics and epilepsy: will the past and present meet? *Epilepsia* 52 1559–1569. 10.1111/j.1528-1167.2011.03203.x 21838793

[B96] Mares P. (2009). Age- and dose-specific anticonvulsant action of bumetanide in immature rats. *Physiol. Res.* 58 927–930. 20059292 10.33549/physiolres.931897

[B97] Markadieu N. Delpire E. (2014). Physiology and pathophysiology of SLC12A1/2 transporters. *Pflugers Arch.* 466 91–105. 10.1007/s00424-013-1370-5 24097229 PMC3877717

[B98] Mazarati A. Shin D. Sankar R. (2009). Bumetanide inhibits rapid kindling in neonatal rats. *Epilepsia* 50 2117–2122. 10.1111/j.1528-1167.2009.02048.x 19260939 PMC2732750

[B99] Mikawa S. Wang C. Shu F. Wang T. Fukuda A. Sato K. (2002). Developmental changes in KCC1, KCC2 and NKCC1 MRNAs in the rat cerebellum. *Dev. Brain Res.* 136 93–100. 10.1016/S0165-3806(02)00345-0 12101026

[B100] Mòdol L. Cobianchi S. Navarro X. (2014). Prevention of NKCC1 phosphorylation avoids downregulation of KCC2 in central sensory pathways and reduces neuropathic pain after peripheral nerve injury. *Pain* 155 1577–1590. 10.1016/j.pain.2014.05.004 24813295

[B101] Morita Y. Callicott J. H. Testa L. R. Mighdoll M. I. Dickinson D. Chen Q. (2014). Characteristics of the cation cotransporter NKCC1 in human brain: alternate transcripts, expression in development, and potential relationships to brain function and schizophrenia. *J. Neurosci.* 34 4929–4940. 10.1523/JNEUROSCI.1423-13.2014 24695712 PMC3972720

[B102] Moult P. J. Lunzer M. R. Trash D. B. Sherlock S. (1974). Use of bumetanide in the treatment of ascites due to liver disease. *Gut* 15 988–992. 10.1136/gut.15.12.988 4448415 PMC1413073

[B103] Munoz A. Mendez P. DeFelipe J. Alvarez-Leefmans F. J. (2007). Cation-chloride cotransporters and GABA-ergic innervation in the human epileptic hippocampus. *Epilepsia* 48 663–673. 10.1111/j.1528-1167.2007.00986.x 17319917

[B104] Murdoch W. R. Auld W. H. (1975). Bumetanide—acute and long term studies of a new high potency diuretic. *Postgrad. Med. J.* 51 10–14. 10.1136/pgmj.51.591.10 1161670 PMC2495680

[B105] Nagelhus E. A. Ottersen O. P. (2013). Physiological roles of aquaporin-4 in brain. *Physiol. Rev.* 93 1543–1562. 10.1152/physrev.00011.2013 24137016 PMC3858210

[B106] NCT00830531 (2017). *Pilot Study of Bumetanide for Newborn Seizures.* Available at: https://clinicaltrials.gov/ct2/show/NCT00830531 (accessed on January 20, 2019).

[B107] NCT01434225 (2015). *NEMO1:NEonatal Seizure Using Medication Off-Patent (NEMO1).* Available at: https://clinicaltrials.gov/ct2/show/NCT01434225 (accessed on January 20, 2019).

[B108] Nicholls J. G. Martin A. R. Fuchs P. A. Brown D. A. Diamond M. E. Weisblat D. A. (2012). *From Neuron to Brain*, 5th Edn. Sunderland, MA: Sinauer Associates.

[B109] Nielsen O. B. Feit P. W. (1978). Structure-activity relationships of aminobenzoic acid diuretics and related compounds (1). *Diuretic Agents* 83 12–23. 10.1021/bk-1978-0083.ch002 1109574

[B110] Nigam S. K. Bush K. T. Martovetsky G. Ahn S. Y. Liu H. C. Richard E. (2015). The organic anion transporter (OAT) family: a systems biology perspective. *Physiol. Rev.* 95 83–123. 10.1152/physrev.00025.2013 25540139 PMC4281586

[B111] Nitta T. Hata M. Gotoh S. Seo Y. Sasaki H. Hashimoto N. (2003). Size-selective loosening of the blood-brain barrier in claudin-5–deficient mice. *J. Cell Biol.* 161 653–660. 10.1083/jcb.200302070 12743111 PMC2172943

[B112] O’Donnell M. E. Duong V. Suvatne J. Foroutan S. Johnson D. M. (2005). Arginine vasopressin stimulation of cerebral microvascular endothelial cell Na-K-Cl cotransporter activity is V1 receptor and [Ca] dependent. *Am. J. Physiol. Cell Physiol.* 289 C283–C292. 10.1152/ajpcell.00001.2005 15800057

[B113] O’Donnell M. E. Lam T. I. Tran L. Q. Foroutan S. Anderson S. E. (2006). Estradiol reduces activity of the blood-brain barrier Na-K-Cl cotransporter and decreases edema formation in permanent middle cerebral artery occlusion. *J. Cereb. Blood Flow Metab.* 26 1234–1249. 10.1038/sj.jcbfm.9600278 16421506

[B114] O’Donnell M. E. Tran L. Lam T. I. Liu X. B. Anderson S. E. (2004). Bumetanide inhibition of the blood-brain barrier Na-K-Cl cotransporter reduces edema formation in the rat middle cerebral artery occlusion model of stroke. *J. Cereb. Blood Flow Metab.* 24 1046–1056. 10.1097/01.WCB.0000130867.32663.90 15356425

[B115] Okabe A. Ohno K. Toyoda H. Yokokura M. Sato K. Fukuda A. (2002). Amygdala kindling induces upregulation of MRNA for NKCC1, a Na+, K+–2Cl- cotransporter, in the rat piriform cortex. *Neurosci. Res.* 44 225–229. 10.1016/S0168-0102(02)00093-7 12354637

[B116] Olsen U. B. (1977). The pharmacology of bumetanide. *Acta Pharmacol. Toxicol.* 41 1–28. 10.1111/j.1600-0773.1977.tb03209.x 331869

[B117] Østby I. Øyehaug L. Einevoll G. T. Nagelhus E. A. Plahte E. Zeuthen T. (2009). Astrocytic mechanisms explaining neural-activity-induced shrinkage of extraneuronal space. *PLoS Comput. Biol.* 5:e1000272. 10.1371/journal.pcbi.1000272 19165313 PMC2613522

[B118] Owens D. F. Kriegstein A. R. (2002). Is there more to gaba than synaptic inhibition? *Nat. Rev. Neurosci.* 3 715–727. 10.1038/nrn919 12209120

[B119] Pace A. J. Lee E. Athirakul K. Coffman T. M. O’Brien D. A. Koller B. H. (2000). Failure of spermatogenesis in mouse lines deficient in the Na(+)-K(+)-2Cl(–) cotransporter. *J. Clin. Investig.* 105 441–450. 10.1172/JCI8553 10683373 PMC289162

[B120] Pacifici G. M. (2012). Clinical pharmacology of the loop diuretics furosemide and bumetanide in neonates and infants. *Pediatr. Drugs* 14 233–246. 10.2165/11596620-000000000-00000 22702741

[B121] Palma E. Amici M. Sobrero F. Spinelli G. Di Angelantonio S. Ragozzino D. (2006). Anomalous levels of Cl- transporters in the hippocampal subiculum from temporal lobe epilepsy patients make GABA excitatory. *Proc. Natl. Acad. Sci. U.S.A.* 103 8465–8468. 10.1073/pnas.0602979103 16709666 PMC1482515

[B122] Parpura V. Basarsky T. A. Liu F. Jeftinija K. Jeftinija S. Haydon P. G. (1994). Glutamate-mediated astrocyte-neuron signalling. *Nature* 369 744–747. 10.1038/369744a0 7911978

[B123] Patyal P. Alvarez-Leefmans F. J. (2016). Expression of NKCC1 and aquaporins 4, 7 and 9 in mouse choroid plexus and ependymal cells. *FASEB J.* 30:lb621 10.1096/fasebj.30.1_supplement.lb621

[B124] Plotkin M. D. Kaplan M. R. Peterson L. N. Gullans S. R. Hebert S. C. Delpire E. (1997a). Expression of the Na(+)-K(+)-2Cl- cotransporter BSC2 in the nervous system. *Am. J. Physiol. Cell Physiol.* 272 C173–C183. 10.1152/ajpcell.1997.272.1.C173 9038823

[B125] Plotkin M. D. Snyder E. Y. Hebert S. C. Delpire E. (1997b). Expression of the Na-K-2Cl cotransporter is developmentally regulated in postnatal rat brains: a possible mechanism underlying GABA’s excitatory role in immature brain. *J. Neurobiol.* 33 781–795. 10.1002/(SICI)1097-4695(19971120)33:6<781::AID-NEU6>3.0.CO;2-5 9369151

[B126] Pressler R. M. Boylan G. B. Marlow N. Blennow M. Chiron C. Cross J. H. (2015). Bumetanide for the treatment of seizures in newborn babies with hypoxic ischaemic encephalopathy (NEMO): an open-label, dose finding, and feasibility phase 1/2 trial. *Lancet Neurol.* 14 469–477. 10.1016/S1474-4422(14)70303-5 25765333

[B127] Puskarjov M. Kahle K. T. Ruusuvuori E. Kaila K. (2014). Pharmacotherapeutic targeting of cation-chloride cotransporters in neonatal seizures. *Epilepsia* 55 806–818. 10.1111/epi.12620 24802699 PMC4284054

[B128] Rahmanzadeh R. Eftekhari S. Shahbazi A. Ardakani M. K. Rahmanzade R. Mehrabi S. (2017). Effect of bumetanide, a selective NKCC1 inhibitor, on hallucinations of schizophrenic patients; a double-blind randomized clinical trial. *Schizophr. Res.* 184 145–146. 10.1016/j.schres.2016.12.002 27956008

[B129] Rahmanzadeh R. Shahbazi A. Ardakani M. K. Mehrabi S. Rahmanzade R. Joghataei M. T. (2016). Lack of the effect of bumetanide, a selective NKCC1 inhibitor, in patients with schizophrenia: a double-blind randomized trial. *Psychiatry Clin. Neurosci.* 71 72–73. 10.1111/pcn.12475 27800670

[B130] Reid A. Y. Riazi K. Campbell Teskey G. Pittman Q. J. (2013). Increased excitability and molecular changes in adult rats after a febrile seizure. *Epilepsia* 54 e45–e48. 10.1111/epi.12061 23293960

[B131] Rivera C. Voipio J. Payne J. A. Ruusuvuori E. Lahtinen H. Lamsa K. (1999). The K+/Cl- Co-transporter KCC2 renders GABA hyperpolarizing during neuronal maturation. *Nature* 397 251–255. 10.1038/16697 9930699

[B132] Römermann K. Fedrowitz M. Hampel P. Kaczmarek E. Töllner K. Erker T. (2017). Multiple blood-brain barrier transport mechanisms limit bumetanide accumulation, and therapeutic potential, in the mammalian brain. *Neuropharmacology* 117 182–194. 10.1016/j.neuropharm.2017.02.006 28192112

[B133] Rutledge E. M. Kimelberg H. K. (1996). Release of [3H]-D-aspartate from primary astrocyte cultures in response to raised external potassium. *J. Neurosci.* 16 7803–7811. 10.1523/JNEUROSCI.16-24-07803.1996 8987808 PMC6579231

[B134] Sansa G. Carlson C. Doyle W. Weiner H. L. Bluvstein J. Barr W. (2011). Medically refractory epilepsy in autism. *Epilepsia* 52 1071–1075. 10.1111/j.1528-1167.2011.03069.x 21671922

[B135] Saunders N. R. Liddelow S. A. Dziegielewska K. M. (2012). Barrier mechanisms in the developing brain. *Front. Pharmacol.* 3:46 10.3389/fphar.2012.00046 PMC3314990 22479246

[B136] Sedmak G. Jovanov-Miloševiæ N. Puskarjov M. Ulamec M. Krušlin B. Kaila K. (2016). Developmental expression patterns of KCC2 and functionally associated molecules in the human brain. *Cereb. Cortex* 26 4574–4589. 10.1093/cercor/bhv218 26428952

[B137] Shimizu-Okabe C. Yokokura M. Okabe A. Ikeda M. Sato K. Kilb W. (2002). Layer-specific expression of Cl- transporters and differential [Cl-]i in newborn rat cortex. *Neuroreport* 13 2433–2437. 10.1097/00001756-200212200-00012 12499844

[B138] Sivakumaran S. Maguire J. (2016). Bumetanide reduces seizure progression and the development of pharmacoresistant status epilepticus. *Epilepsia* 57 222–232. 10.1111/epi.13270 26659482 PMC5487491

[B139] Smith R. A. Martland T. Lowry M. F. (1996). Children with seizures presenting to accident and emergency. *J. Accid. Emerg. Med.* 13 54–58. 10.1136/emj.13.1.54 8821230 PMC1342611

[B140] Song B. Galande A. K. Kodukula K. Moos W. H. Miller S. M. (2011). Evaluation of the PKa values and ionization sequence of bumetanide using 1H and 13C NMR and UV spectroscopy. *Drug Dev. Res.* 72 416–426. 10.1002/ddr.20443

[B141] Song L. Mercado A. Vázquez N. Xie Q. Desai R. George A. L. Jr. (2002). Molecular, functional, and genomic characterization of human KCC2, the neuronal K–Cl cotransporter. *Mol. Brain Res.* 103 91–105. 10.1016/S0169-328X(02)00190-0 12106695

[B142] Steffensen A. B. Oernbo E. K. Stoica A. Gerkau N. J. Barbuskaite D. Tritsaris K. (2018). Cotransporter-mediated water transport underlying cerebrospinal fluid formation. *Nat. Commun.* 9:2167. 10.1038/s41467-018-04677-9 29867199 PMC5986890

[B143] Su G. Kintner D. B. Flagella M. Shull G. E. Sun D. (2002). Astrocytes from Na+-K+-Cl-cotransporter-null mice exhibit absence of swelling and decrease in EAA release. *Am. J. Physiol. Cell Physiol.* 282 C1147–C1160. 10.1152/ajpcell.00538.2001 11940530

[B144] Sun D. (2010). “The ‘Loop’ diuretic drug bumetanide-sensitive Na+-K+-Cl- cotransporter in cerebral ischemia,” in *New Strategies in Stroke Intervention: Ionic Transporters, Pumps, and New Channels*, ed. Annunziato L. (New York, NY: Humana Press).

[B145] Talos D. M. Sun H. Kosaras B. Joseph A. Folkerth R. D. Poduri A. (2012). Altered inhibition in tuberous sclerosis and type IIb cortical dysplasia. *Ann. Neurol.* 71 539–551. 10.1002/ana.22696 22447678 PMC3334406

[B146] Taylor D. C. Falconer M. A. Bruton C. J. Corsellis J. A. (1971). Focal dysplasia of the cerebral cortex in epilepsy. *J. Neurol. Neurosurg. Psychiatry* 34 369–387. 10.1136/jnnp.34.4.369 5096551 PMC493805

[B147] Töllner K. Brandt C. Römermann K. Löscher W. (2015). The organic anion transport inhibitor probenecid increases brain concentrations of the NKCC1 inhibitor bumetanide. *Eur. J. Pharmacol.* 746 167–173. 10.1016/j.ejphar.2014.11.019 25449033

[B148] Töllner K. Brandt C. Töpfer M. Brunhofer G. Erker T. Gabriel M. (2014). A novel prodrug-based strategy to increase effects of bumetanide in epilepsy. *Ann. Neurol.* 75 550–562. 10.1002/ana.24124 24615913

[B149] Traynelis S. F. Dingledine R. (1989). Role of extracellular space in hyperosmotic suppression of potassium-induced electrographic seizures. *J. Neurophysiol.* 61 927–938. 10.1152/jn.1989.61.5.927 2723735

[B150] Tyzio R. Khalilov I. Represa A. Crepel V. Zilberter Y. Rheims S. (2009). Inhibitory actions of the gamma-aminobutyric acid in pediatric sturge-weber syndrome. *Ann. Neurol.* 66 209–218. 10.1002/ana.21711 19743469

[B151] Tyzio R. Nardou R. Ferrari D. C. Tsintsadze T. Shahrokhi A. Eftekhari S. (2014). Oxytocin-mediated GABA inhibition during delivery attenuates autism pathogenesis in rodent offspring. *Science* 343 675–679. 10.1126/science.1247190 24503856

[B152] Urquhart B. L. Kim R. B. (2009). Blood-brain barrier transporters and response to CNS-active drugs. *Eur. J. Clin. Pharmacol.* 65 1063–1070. 10.1007/s00228-009-0714-8 19727692

[B153] Uvarov P. Llano O. Ludwig A. Airaksinen M. Rivera C. (2013). “Multiple roles of KCC2 in the developing brain,” in *Cellular Migration and Formation of Neuronal Connections*, eds Rubenstein J. Rakic P. (Cambridge, MA: Academic Press), 975–998. 10.1016/B978-0-12-397266-8.00190-3

[B154] Uvarov P. Ludwig A. Markkanen M. Pruunsild P. Kaila K. Delpire E. (2007). A novel N-terminal isoform of the neuron-specific K-Cl cotransporter KCC2. *J. Biol. Chem.* 282 30570–30576. 10.1074/jbc.M705095200 17715129

[B155] Uvarov P. Ludwig A. Markkanen M. Soni S. Hübner C. A. Rivera C. (2009). Coexpression and heteromerization of two neuronal K-Cl cotransporter isoforms in neonatal brain. *J. Biol. Chem.* 284 13696–13704. 10.1074/jbc.M807366200 19307176 PMC2679471

[B156] Vibat C. R. Holland M. J. Kang J. J. Putney L. K. O’Donnell M. E. (2001). Quantitation of Na+-K+-2Cl- cotransport splice variants in human tissues using kinetic polymerase chain reaction. *Anal. Biochem.* 298 218–230. 10.1006/abio.2001.5398 11700976

[B157] Walcott B. P. Kahle K. T. Simard J. M. (2012). Novel treatment targets for cerebral edema. *Neurotherapeutics* 9 65–72. 10.1007/s13311-011-0087-4 22125096 PMC3271162

[B158] Walker P. C. Berry N. S. Edwards D. J. (1989). Protein binding characteristics of bumetanide. *Dev. Pharmacol. Ther.* 12 13–18. 10.1159/000480977 2721330

[B159] Walz W. (1987). Swelling and potassium uptake in cultured astrocytes. *Can. J. Physiol. Pharmacol.* 65 1051–1057. 10.1139/y87-166 3621031

[B160] Walz W. (2000). Role of astrocytes in the clearance of excess extracellular potassium. *Neurochem. Int.* 36 291–300. 10.1016/S0197-0186(99)00137-0 10732996

[B161] Wang H. Yan Y. Kintner D. B. Lytle C. Sun D. (2003). GABA-mediated trophic effect on oligodendrocytes requires Na-K-2Cl cotransport activity. *J. Neurophysiol.* 90 1257–1265. 10.1152/jn.01174.2002 12904508

[B162] Wang S. Zhang X. Q. Song C. G. Xiao T. Zhao M. Zhu G. (2015). In vivo effects of bumetanide at brain concentrations incompatible with NKCC1 inhibition on newborn DGC structure and spontaneous EEG seizures following hypoxia-induced neonatal seizures. *Neuroscience* 286 203–215. 10.1016/j.neuroscience.2014.11.031 25463517

[B163] Ward A. Heel R. C. (1984). Bumetanide. A review of its pharmacodynamic and pharmacokinetic properties and therapeutic use. *Drugs* 28 426–464. 10.2165/00003495-198428050-00003 6391889

[B164] Watabe T. Xu M. Watanabe M. Nabekura J. Higuchi T. Hori K. (2017). Time-controllable Nkcc1 knockdown replicates reversible hearing loss in postnatal mice. *Sci. Rep.* 7:13605. 10.1038/s41598-017-13997-7 29051615 PMC5648887

[B165] Watanabe M. Fukuda A. (2015). Development and regulation of chloride homeostasis in the central nervous system. *Front. Cell. Neurosci.* 9:371. 10.3389/fncel.2015.00371 26441542 PMC4585146

[B166] Weinberger D. R. (1988). Schizophrenia and the frontal lobe. *Trends Neurosci.* 11 367–370. 10.1016/0166-2236(88)90060-4 2469198

[B167] Wilkinson C. M. Fedor B. A. Aziz J. R. Nadeau C. A. Brar P. S. Clark J. J. A. (2019). Failure of bumetanide to improve outcome after intracerebral hemorrhage in rat. *PLoS One* 14:e0210660. 10.1371/journal.pone.0210660 30629699 PMC6328169

[B168] Wittner M. Di Stefano A. Wangemann P. Greger R. (1991). How do loop diuretics act? *Drugs* 41 1–13. 10.2165/00003495-199100413-00003 1712711

[B169] Wu Q. Delpire E. Hebert S. C. Strange K. (1998). Functional demonstration of Na+-K+-2Cl-cotransporter activity in isolated, polarized choroid plexus cells. *Am. J. Physiol. Cell Physiol.* 275 C1565–C1572. 10.1152/ajpcell.1998.275.6.C1565 9843718

[B170] Xhima K. Weber-Adrian D. Silburt J. (2016). Glutamate induces blood-brain barrier permeability through activation of N-methyl-D-aspartate receptors. *J. Neurosci.* 36 12296–12298. 10.1523/JNEUROSCI.2962-16.2016 27927949 PMC6601974

[B171] Xu W. Mu X. Wang H. Song C. Ma W. Jolkkonen J. (2017). Chloride co-transporter NKCC1 inhibitor bumetanide enhances neurogenesis and behavioral recovery in rats after experimental stroke. *Mol. Neurobiol.* 54 2406–2414. 10.1007/s12035-016-9819-0 26960329

[B172] Yamada J. Okabe A. Toyoda H. Kilb W. Luhmann H. J. Fukuda A. (2004). Cl(-) uptake promoting depolarizing GABA actions in immature rat neocortical neurones is mediated by NKCC1. *J. Physiol.* 557(Pt 3), 829–841. 10.1113/jphysiol.2004.062471 15090604 PMC1665166

[B173] Yan Y. Dempsey R. J. Sun D. (2001a). Expression of Na+-K+-Cl- cotransporter in rat brain during development and its localization in mature astrocytes. *Brain Res.* 911 43–55. 10.1016/S0006-8993(01)02649-X 11489443

[B174] Yan Y. Dempsey R. J. Sun D. (2001b). Na+-K+-Cl- cotransporter in rat focal cerebral ischemia. *J. Cereb. Blood Flow Metab.* 21 711–721. 10.1097/00004647-200106000-00009 11488540

[B175] Załuska K. Kondrat-Wróbel M. W. Łuszczki J. J. (2018). Comparison of the anticonvulsant potency of various diuretic drugs in the maximal electroshock-induced seizure threshold test in mice. *Adv. Clin. Exp. Med.* 27 609–613. 10.17219/acem/68694 29558036

[B176] Zeuthen T. (2010). Water-transporting proteins. *J. Membr. Biol.* 234 57–73. 10.1007/s00232-009-9216-y 20091162

[B177] Zhang Y. Chen K. Sloan S. A. Bennett M. L. Scholze A. R. O’Keefe S. (2014). An RNA-sequencing transcriptome and splicing database of glia, neurons, and vascular cells of the cerebral cortex. *J. Neurosci.* 34 11929–11947. 10.1523/JNEUROSCI.1860-14.2014. Available at: http://web.stanford.edu/group/barre_lab/brain_rnaseq.html and http://web.stanford.edu/group/barres_lab/brainseq2/brainseq2.html 25186741 PMC4152602

